# Effects of trichothecene production by *Trichoderma arundinaceum* isolates from bean-field soils on the defense response, growth and development of bean plants (*Phaseolus vulgaris*)

**DOI:** 10.3389/fpls.2022.1005906

**Published:** 2022-11-14

**Authors:** Rosa E. Cardoza, Sara Mayo-Prieto, Natalia Martínez-Reyes, Susan P. McCormick, Guzmán Carro-Huerga, M. Piedad Campelo, Álvaro Rodríguez-González, Alicia Lorenzana, Robert H. Proctor, Pedro A. Casquero, Santiago Gutiérrez

**Affiliations:** ^1^ University Group for Research in Engineering and Sustainable Agriculture (GUIIAS), Area of Microbiology, Universidad de León, Ponferrada, Spain; ^2^ University Group for Research in Engineering and Sustainable Agriculture (GUIIAS), Area of Crop Production, Universidad de León, León, Spain; ^3^ Mycotoxin Prevention and Applied Microbiology Research Unit, National Center for Agricultural Utiization Research, Agriculture Research Service, U.S. Department of Agriculture, Peoria, IL, United States

**Keywords:** biological control, gene-clusters, metabolomics, plant-fungal interaction, secondary metabolites, sesquiterpenes, transcriptomics

## Abstract

The trichothecene toxin-producing fungus *Trichoderma arundinaceum* has potential as a biological control agent. However, most biocontrol studies have focused only on one strain, IBT 40837. In the current study, three *Trichoderma* isolates recovered from bean-field soils produced the trichothecene harzianum A (HA) and trichodermol, the latter being an intermediate in the HA biosynthesis. Based on phylogenetic analysis, the three isolates were assigned to the species *T. arundinaceum*. Their genome sequences had a high degree of similarity to the reference IBT 40837 strain, in terms of total genome size, number of predicted genes, and diversity of putative secondary metabolite biosynthetic gene clusters. HA production by these bean-field isolates conferred significant *in vitro* antifungal activity against *Rhizoctonia solani* and *Sclerotinia sclerotiorum*, which are some of the most important bean pathogens. Furthermore, the bean-field isolates stimulated germination of bean seeds and subsequent growth of above ground parts of the bean plant. Transcriptomic analysis of bean plants inoculated with these *T. arundinaceum* bean-field soil isolates indicated that HA production significantly affected expression of plant defense-related genes; this effect was particularly significant in the expression of chitinase-encoding genes. Together, these results indicate that *Trichoderma* species producing non-phytotoxic trichothecenes can induce defenses in plants without negatively affecting germination and development

## 1 Introduction

The common bean (*Phaseolus vulgaris* L.) is the third most important food legume worldwide, surpassed only by soybean [*Glycine max* (L.) Merr.] and peanut (*Arachis hypogaea* L.). Among southern countries of the European Union, Spain, Italy, and Greece are the main producers of this legume. Within Spain, the province of León is the main bean-producer by quantity and quality, accounting for almost 45% of Spanish bean production in 2018 (Ministerio de Agricultura Pesca y Alimentación, 2019). Socio-economic conditions of the province have enabled maintenance of local varieties in traditional, small-scale cropping systems (Casquero et al., 2006). The high quality of this legume has been awarded with a Protected Geographic Indication (PGI) named *Alubia de La Bañeza-León* (EC Reg. n. 256/2010 published on 26 March 2010, OJEU L880/17) (**Figure S1**). In recent years, however, the yield of dry beans in León have been relatively low due, in part, to diseases caused by phytopathogenic fungi and oomycetes (Mayo-Prieto et al., 2015).

Bean crops are susceptible to numerous fungal and oomycete root pathogens, including species of *Fusarium, Rhizoctonia, Sclerotinia*, *Thielaviopsis, Aphanomyces* and *Pythium*. Several of these pathogens can occur as a disease complex (APS, 2010). In the province of León, one of the most frequent bean diseases is “Rhizoctonia root rot” (Valenciano et al., 2006). Several studies have estimated that this disease, together with other related root rots and grey and white molds, can reduce bean yield in León by 50% (Mayo-Prieto et al., 2015).

Over the last two decades, the need for more sustainable strategies to control bean diseases has focused on biological control agents (BCA). *Trichoderma* spp. are considered among the most promising and frequently studied BCAs (Benitez et al., 2004). In the case of bean diseases, some *Trichoderma* species have been shown to inhibit development of *Rhizoctonia solani* and to reduce the effects of this pathogen on plant growth (Mayo-Prieto et al., 2015; Mayo-Prieto et al., 2020a). Furthermore, *Trichoderma* species have also been used to control nematode and insect pests (Goswami et al., 2008; Rodríguez-González et al., 2017).

In addition to the production of extracellular hydrolytic enzymes (e.g., proteases, cellulases, chitinases, glucanases), *Trichoderma* spp. produce a plethora of secondary metabolites (SMs) that are diverse in structure and biological activity. These metabolites include peptaibols, lactones, nitriles, polyketides, non-ribosomal peptides, alkaloids, and terpenes (Sivasithamparam and Ghisalberti, 1998; Hermosa et al., 2014). Among the *Trichoderma* SMs, trichothecenes are a family of approximately 200 toxic sesquiterpenoids that have attracted significant attention in recent years. They are produced by some species in multiple fungal genera, including *Fusarium*, *Isaria*, *Microcyclospora*, *Myrothecium*, *Peltaster*, *Spicellum*, *Stachybotrys*, *Trichoderma* and *Trichothecium* (Proctor et al., 2018; Proctor et al., 2020). Trichothecenes inhibit eukaryotic protein synthesis (Cundliffe et al., 1974; McLaughlin et al., 2009), and some are frequent contaminants of crops used for food and animal feed (O´Donnell et al., 2018). *Stachybotrys* trichothecenes have been implicated in the harmful effects of mold growth in damp buildings on human health (Straus, 2009). Some trichothecene analogs induce apoptosis (Okumwai et al., 1999), some are phytotoxic, and some act as virulence factors in certain plant-*Fusarium* interactions (Harris et al., 1999). Many trichothecene-producing fungi are plant pathogens, and some, such as *Isaria tenuipes*, are insect pathogens. Some *Fusarium* trichothecenes are among the mycotoxins of most concern to food and feed safety (Desjardins, 2006). In contrast, *in vivo* studies indicate that trichothecenes produced by *Trichoderma arundinaceum* and *T. albolutescens* lack phytotoxicity and contribute to the biological control activity against some plant diseases caused by fungi or viruses (Malmierca et al., 2012; Malmierca et al., 2016; Ryu et al., 2017). *Trichoderma arundinaceum* trichothecenes, e.g., harzianum A (HA), induce expression of plant genes related to salicylate (SA) and jasmonate/ethylene (JA)/(ET) defense pathways (Malmierca et al., 2012; Malmierca et al., 2013). In addition, HA exhibits strong antifungal activity against several important phytopathogenic fungi (Malmierca et al., 2012; Malmierca et al., 2013). Trichothecenes produced by *T. albolutescens* (trichodermin and 16-OH-trichodermin) have a protective effect against diseases of tobacco and pepper caused by pepper mottle virus (Ryu et al., 2017).

In addition to the important roles that *Trichoderma* trichothecenes play in the biological activities of the producing species, they also help to maintain the levels of terpene intermediates, e.g., farnesol, squalene, and ergosterol, needed to ensure normal fungal growth and development (Lindo et al., 2018; Lindo et al., 2019; Gutiérrez et al., 2020).

The current study represents a novel approach to recover trichothecene-producing isolates of *Trichoderma* from soils in crop fields. In the present case, these strains were isolated from bean fields located in a region of northwestern Spain with the PGI designation *Alubia de La Bañeza-León*. The selected strains were identified and analyzed using a combination of microbiomics, metabolomics, and comparative genomic techniques. Further, we examined the ability of the resulting isolates to inhibit the growth of the pathogens *Rhizoctonia solani* and *Sclerotinia sclerotiorum*, and also analyzed the effects of the isolates on growth and defense response of bean plants. Finally, a transcriptomic study was carried out to assess the effects of HA on expression of defense-related genes in bean.

## 2 Materials and methods

### 2.1 Strains used and growth conditions

Pathogens *Rhizoctonia solani* R43 and *Sclerotinia sclerotiorum* S47 used for antifungal studies were obtained from the “Pathogens and Antagonists’ collection at the Laboratory for Diagnosis of Pest and Diseases” (University of León, Spain). They were isolated from bean plants grown in a region of the Province of León with the PGI designation of *Alubia de La Bañeza-León* (Mayo-Prieto et al., 2020b). IBT 40837 (Ta37) is a *T. arundinaceum* wild-type strain obtained from the fungal culture collection at the Danish Technical University. Strain Δtri5.3 (=ΔT5.3) is a previously described mutant that was generated by deleting the trichothecene biosynthetic gene *tri5* in strain IBT 40837 (Gutiérrez et al., 2021). *tri5* encodes a terpene synthase (trichodiene synthase) that catalyzes the first commitment step in trichothecene biosynthesis (Malmierca et al., 2013; Gutiérrez et al., 2021). As a result, the deleted *tri5* gene in strain Δtri5.3 precludes it from producing harzianum A or any trichothecene biosynthetic intermediates (Gutiérrez et al., 2021).

All fungal strains were grown in potato dextrose agar medium (PDA) (Difco, Becton Dickinson) and incubated at 28°C in the dark. Strains were usually grown for 7 days. However, for some experiments, e.g., antifungal assays, the incubation period was extended as indicated below.

### 2.2 *Trichoderma* strains isolation and identification

Soil samples were obtained from seven locations within the PGI *Alubia de La Bañeza-León* (**Figure S1**). Five g of soil from each sample, collected at a depth of 10 cm, were processed as previously described (Restovich et al., 2012; Mayo-Prieto et al., 2015; Mayo-Prieto et al., 2020a). Putative *Trichoderma* isolates were detected on Rose Bengal-chloramphenicol-agar medium (Oxoid, Hampshire, UK) based on morphological characteristics (Mayo-Prieto et al., 2015). The isolates were transferred to PDA plates and sporulated for further characterization.

For fungal identification, a small fraction of PDA-grown mycelium from each fungal isolate was resuspended in 100 μL of the reactive PrepMan Ultra (Applied Biosystems), heated to 100 °C for 10 min and then kept at room temperature for 2 min. Debris was removed by centrifugation for 2 min. Finally, 1 μL of the supernatant was used in the PCR reaction using primers corresponding to the ITS5 (5´-GGAAGTAAAAGTCGTAACAAGG-3´) and ITS4 (5´-TCCTCCGCTTATTGATATGC-3´). PCR was carried out using a GeneAmp PCR 2700 system (Applied Biosystems), and each reaction was performed using the DreamTaq DNA polymerase kit (Thermo Scientific) following the manufacturer´s instructions. After electrophoresis, amplicons were visualized by agarose gel electrophoresis, purified using the NucleoSpin Gel and PCR Clean-up systems (Macherey-Nagel), and then sequenced using the BigDye Terminator v3.1 Cycle kit and an ABI 3130x1 automatic sequencer (Applied Biosystems). For fungal identification, amplified ITS sequences were compared to sequences in the non-redundant GenBank NCBI database (http://www.ncbi.nlm.nih.gov) using the BLASTn program (http://www.ncbi.nlm-.nih.gov/BLAST). To refine the preliminary identification, sequences that yielded the highest scores with *Trichoderma* species belonging to the Brevicompactum clade (Kubicek et al., 2019; Gutiérrez et al., 2021) were compared to an in-house database consisting of genome sequences from 35 *Trichoderma* species that correspond to all known trichothecene-producing *Trichoderma* species and some of their closest relatives (Gutiérrez et al., 2021).

### 2.3 Detection, quantification of harzianum A (HA)

To assess HA production, the *Trichoderma* strains selected on the basis of previously described criteria were grown in 250 mL flasks containing 50 mL of complex-malt broth (CM) [0.5% malt extract (Cultimed, Panreac Applichem), 0.5% yeast extract (Difco, Becton Dickinson), 0.5% glucose (Panreac Applichem)], inoculated with 2 x 10^6^ spores/mL and incubated for 48 h at 28°C and 250 rpm in an orbital shaker. 10 mL of this preinoculum were then transferred to a new 250 mL flask containing 50 mL of potato-dextrose broth (PDB) (Difco, Becton Dickinson), and were incubated at 28°C and 250 rpm for 48 h. HA was purified and quantified from the culture supernatants as described by Cardoza et al. (2011). In summary, twenty microliters of the 1:5 diluted culture broth extract were applied to a Waters analytical column (YMC™) of 150 x 4.6 mm and eluted with a 1 mL/min flux of mobile phase [(60% water plus 0.1% trifluoroacetic acid)/40% acetonitrile], for 30 min, reaching 100% of acetonitrile in 10 min, held for 5 min, and then returned to the initial conditions (total time of the program= 50 min). Using these conditions HA eluted at approximately 24 min. Quantification of HA was carried out by comparison of that 24 min peak area against a calibration curve made with known amounts of purified HA (Cardoza et al., 2011). Each quantification was performed in duplicate.

### 2.4 Detection of trichothecene intermediates and aspinolides by gas chromatography-mass spectrometry (GC-MS)

We used GC-MS to detect other trichothecene intermediate compounds, e.g., trichodermol, trichodiene, isotrichodiol, 12,13-epoxytrichothec-9-ene (=EPT, trichothecene) (Cardoza et al., 2011), and other secondary metabolites also known to have antifungal activity, e.g., aspinolides. TP6.6 strain was grown in liquid yeast extract-peptone-dextrose (YEPD) cultures [5% glucose, 0.1% yeast extract, 0.1% peptone (Difco, Becton Dickinson)] (20 mL YEPD in 50 ml Erlenmeyer flasks) at 200 rpm and 28°C or 20°C, which are optimal and suboptimal temperatures, respectively, for trichothecene production. After 7 days, cultures were extracted with 3 mL of ethyl acetate. One μL of the extract was injected into a Hewlett Packard 6890 gas chromatograph fitted with a HP-5MS column (30 m, 0.25 mm, 0.25 µm) and a 5973-mass detector. The carrier gas was helium with a 20:1 split ratio and a 20 mL/min split flow. The column was held at 150°C for one minute following injection, heated to 280°C at 30°C/min and held for 3.6 min for a total run time of 9 min. Compound identifications were based on GC-MS comparisons with purified standards.

### 2.5 Fungal confrontation and antifungal assays on membranes

These assays were performed as described in previous reports (Malmierca et al., 2013) with some differences. For example, for the antifungal assays there were used only cellophane membranes (Shengzhou Pengyu Trading Co., Ltd) and pathogen´s growth was monitored until the third week of incubation at 28°C. Similarly, fungal confrontation assays were also monitored for three weeks, to see the long-term effects of the confrontation on the ability of the *Trichoderma* strains to overgrow the pathogens.

### 2.6 Fungal DNA extraction for genome sequencing

Spores (1 x 10^6^ spores/mL) of the fungal strains isolated during the current study were grown in CM medium for 2 days at 28°C with shaking at 200 rpm. Mycelia were harvested by filtration, freeze dried, ground to a powder, and genomic DNAs were extracted using the DNeasy Plant Mini Kit (QIAGEN, Hilden, Germany). DNA purity and concentration were determined in a Nanodrop ND-1000 spectrophotometer.

### 2.7 Genome sequences and analysis

The genome sequence from strain TP6.6 was generated using a MiSeq Illumina platform (Illumina, Inc.), and was assembled with CLC NGS Cell v. 9.5 (Qiagen, Redwood City) as previously described (Proctor et al., 2018). For TP15.11 and TP19.13, their genome sequences were generated by Macrogen, Inc. (Seoul, Korea; https://dna.macrogen.com) using an Illumina platform, and sequence assemblies were generated using the SPAdes (v3.15.0) assembler software (Bankevich et al., 2012). The assembled but unannotated genome sequences were deposited into NCBI’s GenBank database with accession numbers JAMPTP000000000 (TP6.6), JAMQRS000000000 (TP15.11), and JAMQTF000000000 (TP19.13).

Assembled genomes were analyzed to detect secondary metabolite gene clusters with the prediction and annotation tool Augustus (Stanke et al., 2006; Hoff and Stanke, 2013), and Blast2GO software (Conesa et al., 2005). Then, the predicted genes/proteins were analyzed by Blast software using the OmicsBox v. 2.1.14 package (BioBam Informatics, https://www.biobam.com/omicsbox). Finally, the Augustus annotated genomes were analyzed with the antiSMASH software version 3.0.5 to detect putative secondary metabolite gene clusters (Medema et al., 2011).

### 2.8 Phylogenetic analysis

A *Trichoderma* species tree was inferred using amino acid sequences deduced from coding sequences of five housekeeping genes [*act1* (encoding actin); *fas1* (fatty acid synthase alpha subunit); *rpb2* (RNA polymerase 2nd largest subunit), *lcb2* (sphinganine palmitoyl transferase subunit 2); and *tef1* (translation elongation factor 1-alpha)], which were retrieved from the genome sequences of TP6.6, TP15.11, and TP19.13 isolates. In this analysis, amino acid sequences of the five genes were also retrieved from previously generated genome sequences of 35 other *Trichoderma species* Gutiérrez et al. (2021). Finally, protein sequences were aligned using the software MUSCLE as implemented in MEGAX (Kumar et al., 2021), and then the alignments were concatenated using Sequence Matrix Software (Vaidya et al., 2011). The concatenated alignment was subjected to a maximum likelihood analysis (ML) using the software IQ-Tree, version 1.6.7. (Nguyen et al., 2015). Branch support was determined by bootstrap analysis using 1,000 pseudoreplicates. In addition, to assess the consistency of trees inferred from the five housekeeping genes, a gene concordance factor (GCF) analysis was performed as described by Minh et al., 2020. Thus, the percentage of individual protein trees containing each of the branches was calculated and included in the species tree. For the GCF analysis, a second concatenated-partitioned tree was constructed by selecting for each amino acid sequence alignment, the best-fit model of evolution deduced from the previous IQ-Tree analysis as described by Gutiérrez et al. (2021). This concatenated-partitioned alignment was also subjected to a ML analysis as implemented in IQ-Tree. The resulting IQ-tree file was used for GCF calculation based on Minh et al., 2020. In addition, an ML tree was inferred from *tri5* coding sequences from selected *Trichoderma* isolates recovered from bean-field soils and previously described *Trichoderma*-*tri5* sequences. Sequences alignment and ML analysis were performed as indicated above.

### 2.9 Microbiome analysis from soils

One of the soil samples that contained trichothecene-producing *Trichoderma* spp. was further used for a detailed microbiome analysis. This analysis was performed by Biome Makers Inc. (BECROP Smart Agriculture, Biome Makers, Europe. 47006 Valladolid, Spain) (https://biomemakers.com), through the use of the BeCrop^®^ patented Technology. Four different samples were collected in sterile tubes from different points of the selected plot at two different time points (June-2019 and September-2019) at a depth of between 0 and 10 cm, which is a similar strategy to that followed to collect all samples used in this work. Soil samples were then analyzed by the Biome Makers laboratory.

#### 2.9.1 DNA extraction, PCR amplification, and DNA sequencing

DNAs from these four samples was extracted using the Powerlyzer Powersoil Kit (Qiagen) by the BeCrop^®^ Platform (patent publication number: WO2017096385, Biome Makers) (Becares and Fernández, 2017). For the identification of the fungal microbial communities associated with these bulk and rhizosphere soil samples, internal transcribed spacer (ITS) marker regions were amplified with Biome Makers custom primers (patent WO2017096385) (Gobbi et al., 2022). All PCR reactions were carried out following rutinary procedures, and libraries were prepared following a two-step PCR illumina protocol (Gobbi et al., 2019; Liao et al., 2019), and they were subsequently sequenced on an Illumina MiSeq device (Illumina) using 2 x 301 paired-end reads.

#### 2.9.2 Bioinformatic processing

Primers were removed from paired end reads using Cutadapt (Martin, 2011). Then the trimmed reads were merged with a minimum overlapping of 100 nucleotides. Next, the sequences were quality filtered by Expected Error analysis (Edgar and Flyvbjerg, 2015) with a maximum value of 1.0. After quality pre-processing, reads having single nucleotide differences were iteratively clustered together to form Amplicon Sequencing Variants (ASVs) using Swarm (Mahé et al., 2021). *De novo* chimeras and remaining singletons were subsequently removed (Edgar et al., 2011). Finally, the ASVs were compared against Biome Makers´s internal reference database of amplicons using a global alignment with 97% identity to select the best hit; in cases of multiple best hits with identical qualities, the pipeline automatically adjusted the ASV result resolution to the nearest common ancestor of the hits’ taxonomies, which can decrease it to genus or family level. The reference database of amplicons was built using internal manually curated taxonomies from the latest version available of UNITE 8.3 for ITS sequences (Glöckner et al., 2017; Nilsson et al., 2019).

### 2.10 Growth of *P. vulgaris*, inoculation with *Trichoderma* spores, and infection with *R. solani* R43 and *S. sclerotiorum* S47

Bean seeds of variety “Riñón” harvested in 2019 and without visible disease symptoms were selected for this assay. The seeds were surface disinfested by washing with tap water, submerging for 3 minutes in a 1% bleach solution, and rinsing for 6 minutes with sterile distilled water as described previously (Mayo-Prieto et al., 2015).

Bean plants were grown in 1L pots, which were filled with sterilized (autoclaved for 1h, 120°C and a pressure of 1kg/cm^2^, for three consecutive days) peat substrate (Typ3, TYPical, Brill Substrate GmbH & Co) and surface irrigated with tap water (250 mL). Typ3 substrate has high air capacity and drainage, with a fine structure, pH 5.7, and elaborated from 65% white peat, and 35% black peat, and containing 0.5 kg/m^3^ NPK as fertilizer.

Fungal pathogens (*R. solani* R43 and *S. sclerotiorum* S47) were grown on 90 mm PDA Petri dishes by incubation at 25°C for 16 days in an incubation chamber. After this period, a liquid suspension of these fungal mycelia was prepared in sterilized distilled water as follows: PDA culture medium from 5 plates was mixed with 10 mL of water/plate, added to 1 L of distilled water and grounded to homogeneity. A suspension was prepared using PDA medium from 5 uninoculated plates to be used as a control. 50 mL of the homogenized pathogen suspension were added to each pot on the substrate surface, the inoculum was then covered by a thin layer of substrate to cover the pathogens inoculum and the pots were maintained for 8 days in the growth chamber at 25°C/16°C (16h/8h) in the dark.

Seeds were coated with a conidial suspension as described previously (Mayo-Prieto et al., 2015). Briefly, spore suspensions were prepared at a final concentration of 2 × 10^7^ spores/mL in germination buffer (20 mM glucose, 20 mM KH_2_PO_4_). Bean seeds were surface sterilized (1% sodium hypochlorite for 3 min and distilled water for 6 min) and coated with the spore suspension of each *Trichoderma* isolate. The seeds were submerged in the spore suspension (45 seeds per 20 mL spores suspension) and they were dried in a flow chamber for 12 h. One inoculated “Riñón” bean, or one control bean without *Trichoderma* or without the pathogen, was placed in each pot, under the following treatments: 1- Control; 2- Δtri5.3; 3- TP6.6; 4- TP19.13. Pots were kept in a growth chamber for 45 days at 25°C (16 h)- light and 16 °C (8 h)- dark, 60% relative humidity. Pots were irrigated with tap water after the 9^th^ day after sowing, and from that time, 2 irrigations were performed per week using 250 mL/pot. In addition, 250 mL/pot of a water dissolved nutritive solution (15-5-30/N-P-K, Hakaphos^®^, Compo Expert Spain) was applied on the 19^th^ and 33^rd^ day after sowing.

To avoid cross contamination, pots with and without the pathogen were placed on tables separated for 1 m.

The effect of the treatment with each *Trichoderma* strain on germination and plant growth, as well as on the plant response against *R. solani* and *S. sclerotiorum* were evaluated. Leaf samples were detached from four plants per treatment, infected or not with these pathogens for each *Trichoderma* strain, including controls without *Trichoderma* or without pathogens. Leaves were then maintained at -80°C for further analysis. Also, entire plants were washed to remove the potting mixture to detect any phenotypic differences in growth of aerial and below ground parts of the plants in the different treatments.

### 2.11 Statistical analysis of plant parameters

Means and statistical errors of the recorded data were calculated to evaluate bean germination and development for each treatment (three *Trichoderma* spp. and two pathogens). These bean parameters were analyzed by Levene’s test and compared by analysis of variance (two-ways ANOVA) for a completely randomized design including main effects of *Trichoderma* isolates (with three levels: Δtri5.3, TP6.6 and T19.13), and pathogens (with two levels: *R. solani* R43 and *S. sclerotiorum* S47). Finally, a *post-hoc* analysis of Duncan`s test was performed for each *Trichoderma* isolate and pathogen.

### 2.12 RNAseq and real-time quantitative PCR (qPCR) analysis

Leaves detached from plants of the different treatments were used for RNA extraction. Two batches of leaves were used per treatment, each with leaves from four different plants, which were independently processed, and constitute two biological replicates for each condition/treatment. Leaves were ground to a powder in a ceramic mortar, under liquid nitrogen. RNAs were extracted using TRIZOL reagent (Invitrogen) as described previously (Lindo et al., 2020). After the extraction procedure, RNA was treated with RNase-free DNase and purified through a Zymo-Spin column (Zymo Research). Total RNAs from each sample (=treatment) were quantified and the integrity was qualified by a NanoDrop (Thermo Fisher Scientific Inc.) and an Agilent 2100 Bioanalyzer (Agilent Technologies), respectively.

#### 2.12.1 RNAseq analysis

Total RNAs with RNA integrity (RIN) values above 6.5 were sent to Macrogen, Inc. (https://dna.macrogen.com) for cDNA synthesis, library preparation, sequencing, and assembly. In this process, library construction and cDNAs sequencing were carried out using the TruSeq^®^ Stranded mRNA LT Sample Prep kit on an Illumina platform. Trimmed reads were obtained by Trimmomatic (Bolger et al., 2014) software to remove adapter sequences, and were mapped to the reference *Phaseolus vulgaris* transcripts database, retrieved from NCBI repository (https://ftp.ncbi.nlm.nih.gov/genomes/genbank/plant/Phaseolus_vulgaris) (Latest assembly date 2022-04-06), with HISAT2 (Kim et al., 2019), splice-aware aligner. After the read mapping, Stringtie (Pertea et al., 2015) was used for transcript assembly. Expression profile was calculated for each sample and transcript/gene as reads count, FPKM (Fragments per kilobase of transcript per million mapped reads) and TPM (Transcripts per million reads).

DEG (Differentially Expressed Genes) analyses were performed on the nine designed comparison pairs. Thus, reads count data were normalized estimating size factors, and using them, the resulting count data were normalized with the Trimmed mean of M-values (TMM) method in edgeR library (Robinson et al., 2010). Then, a statistical test was performed with the normalized data using TMM count method for exact test (exactTest) in edgeR. Those transcripts showing a differential expression value/fc/>= 2 and a p-value <0.05 were considered as differentially expressed in the compared conditions.

For DEG analysis the *Phaseolus vulgaris* genes and proteins were functionally annotated using the application Blast2Go included in the software package OmicsBox (BioBam Informatics S.L).

#### 2.12.2 qPCR analysis

This analysis was used to validate RNAseq data. RNA samples used for the qPCR analysis were the same as those used for RNAseq analysis. Thus, there were used samples from two biological replicates. cDNAs were synthesized separately from each replicate, and after the synthesis, these two cDNAs were mixed and used as a unique sample from each condition. qPCR reactions were carried out from these “mixed” samples as described previously (Lindo et al., 2020), and were analyzed by the 2^-ΔΔCT^ method (Livak and Schmittgen, 2001), using the actin gene as a housekeeping internal control, and bean plants not inoculated with *Trichoderma* as the reference sample. The arithmetic average, and the standard deviation for three technical replicates were represented.

All oligonucleotides used were stable under the conditions used in the qPCR study as deduced from melting temperature study carried out for each oligonucleotide pair at the end of the 40 qPCR amplification cycles.

## 3 Results

### 3.1 Isolation of fungal strains from bean crop soils

Twenty-eight soil samples were collected from fields that were planted with beans at the time of sampling or during the previous cropping season. The fields have been managed using organic farming procedures and were located in seven municipalities within the PGI *Alubia de La Bañeza-León*, which is located in the northwestern Spanish province of León. The municipality names were as follows: Felechares de la Valdería (1 sample), Matalobos del Páramo (11 samples), Santibáñez de la Isla (3 samples), Urdiales del Páramo (2 samples), Villabúrbula (8 samples), Villamor de Órbigo (1 sample, Bustillo del Páramo (1 sample)), and Villarejo de Órbigo (1 sample) (**Figures S1A, B**).

**Figure 1:**
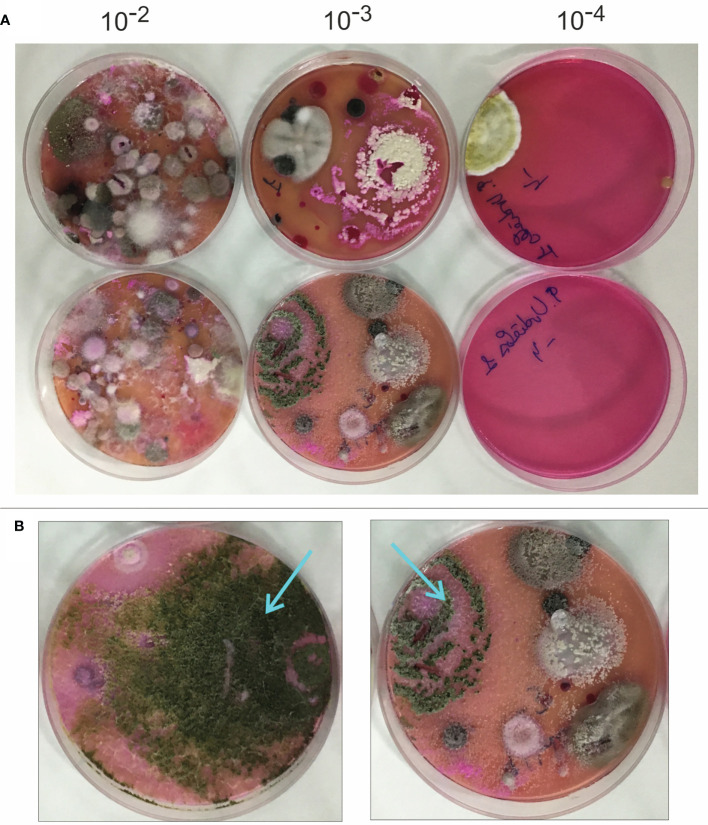
Isolation of *Trichoderma* strains from bean crop soils. **(A)** aspect of colonies isolated in Rose Bengal-Chloramphenicol medium used for fungal growth from dilutions 10^-2^ to 10^-4^ of the different bean crop soils analyzed in this work. **(B)** Two *Trichoderma* colonies are pointed to show the characteristic shape of most of the colonies from the species belonging to the genus *Trichoderma* that have been isolated in the current study.

The soil samples were processed as described in the methods section, by cultivating serial dilutions (10^-2^ to 10^-4^) in Rose Bengal-chloramphenicol agar medium, and incubated at 15°C for two weeks under natural light conditions to allow the growth of fungal colonies (**Figure 1A**). Colonies exhibiting *Trichoderma*-like morphology (**Figure 1B**) (i.e., green-spored colonies, growing as concentric sporulated green rings, and light pink-colored in the reverse side of the plate), were selected and identified as *Trichoderma* by analyzing their ITS sequences.

### 3.2 Identification of *Trichoderma* species among all the fungal isolates

A total of 328 fungal colonies were selected from the Rose Bengal-Chloramphenicol plates and transferred to PDA medium. After incubation for 7 days at 28°C, 113 of these colonies were selected, based on their *Trichoderma*-like colony morphology (see **Figure 1**), for a preliminary identification using the DNA sequence of their ITS1 region. The identification was done by comparison of PCR-amplified sequences from each isolate with DNA sequences in NCBI’s nonredundant database. The results of this analysis indicated that 85 isolates were members of the genus *Trichoderma* and that the remaining isolates were members of other fungal genera, including *Fusarium (n=17), Penicillium (3), Clonostachys (3), Mucor (2), Chaetomium (1)*, and *Papulaspora (1)* (**Table S1**).

### 3.3 Determination of species identity and selection of *Trichoderma* spp. with potential to produce trichothecenes

**Figure 2:**
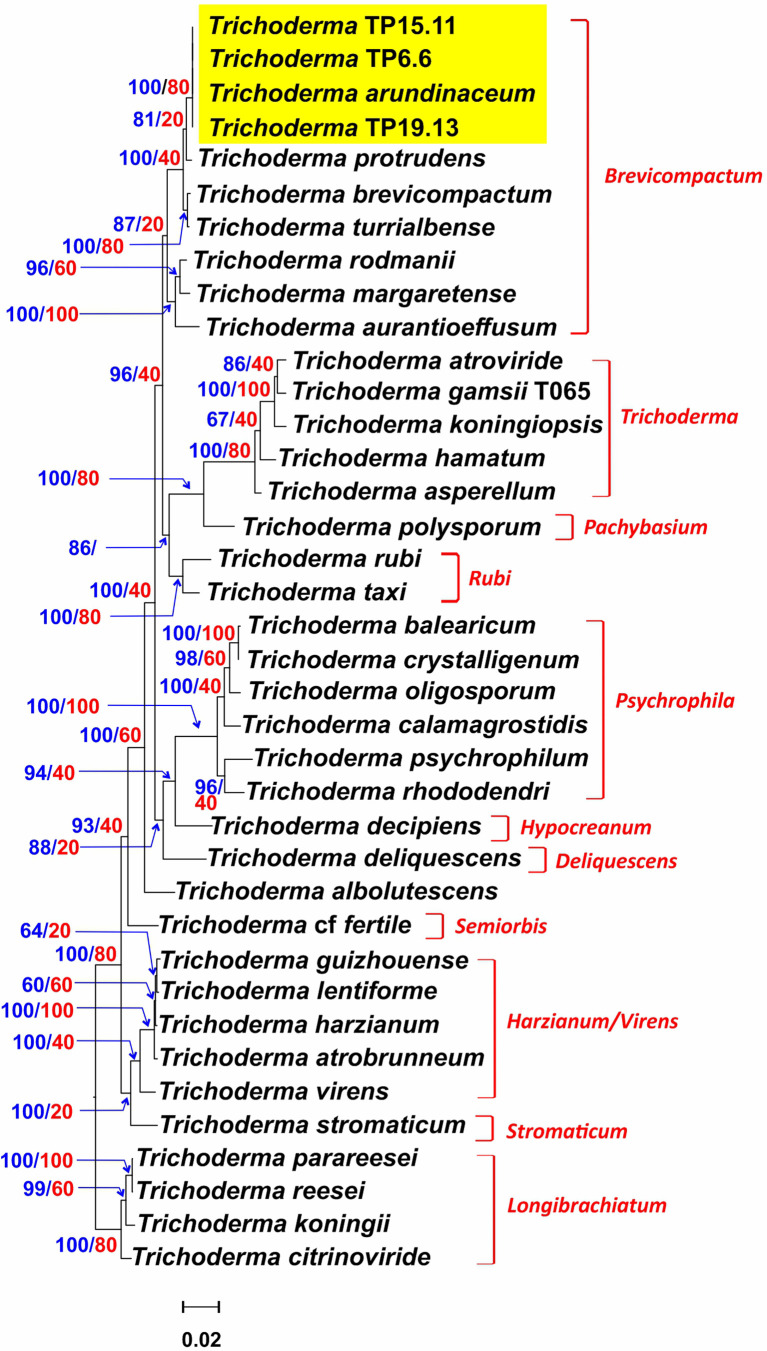
*Trichoderma species* tree inferred by maximum likelihood (ML) analysis of full-length amino acid sequences deduced from the full-length coding regions of five housekeeping genes. Numbers on branches are bootstrap values (blue type) based on 1000 pseudoreplicates and gene concordance factors (GCF, red type). Lineage names, as described previously (Kubicek et al., 2019; Gutiérrez et al., 2021), are indicated in red type to the right of the tree. Yellow rectangle highlights the positions of the three isolates recovered during the current study.

Of the 85 putative *Trichoderma* isolates, only TP6.6, TP6.13, TP15.11 and TP19.13 had ITS1 sequences that were more closely related to sequences from members of clade *Brevicompactum* than to members of other *Trichoderma* clades. Isolates TP6.6 and TP6.13 were both recovered from soil sample number 6 and had identical ITS1 sequences. Therefore, we selected TP6.6 for further analyses and did not examine TP6.13 further. The determination of species identity of TP6.6, TP15.11 and TP19.13 was done by phylogenetic analysis of the predicted amino acid sequences of five housekeeping genes (*act1, fas1*, *rpb2*, *lcb2*, and *tef1*) that were retrieved from the genome sequence data of the three isolates. The full-length protein sequences were aligned with the orthologous sequences from 35 *Trichoderma* species representing a wide breadth of the known phylogenetic diversity within the genus. In maximum likelihood trees inferred from sequences from four of five individual genes, the three bean-field isolates formed an exclusive and well-supported clade with the reference *T. arundinaceum* strain, IBT 40837. In a tree inferred from concatenated alignments of all five genes, the bean field isolates also formed an exclusive and well-supported (100% bootstrap value) with the reference *T. arundinaceum* strain (**Figure 2**). These findings indicate that isolates TP6.6, TP15.11 and TP19.13 are *T. arundinaceum*.

The rationale to identify isolates of the clade *Brevicompactum* was that previous studies indicated that most species in this lineage produce trichothecenes (Gutiérrez et al., 2021). Furthermore, according to the ITS1 sequence analysis, none of the *Trichoderma* isolates recovered in the current study belonged to the clades *Psychrophila* or *Rubi*, two other lineages in which most species produce trichothecenes (Chen et al., 2018; Gutiérrez et al., 2021). In addition, the ITS1 analysis indicated that none of the isolates belonged to species that produce trichothecenes but have not been assigned to a multispecies *Trichoderma* clade (e.g., *T. albolutescens*) (Ryu et al., 2017; Chen et al., 2021; Gutiérrez et al., 2021).

#### 3.3.1 Detection and quantification of harzianum A (HA) by HPLC

**Figure 3:**
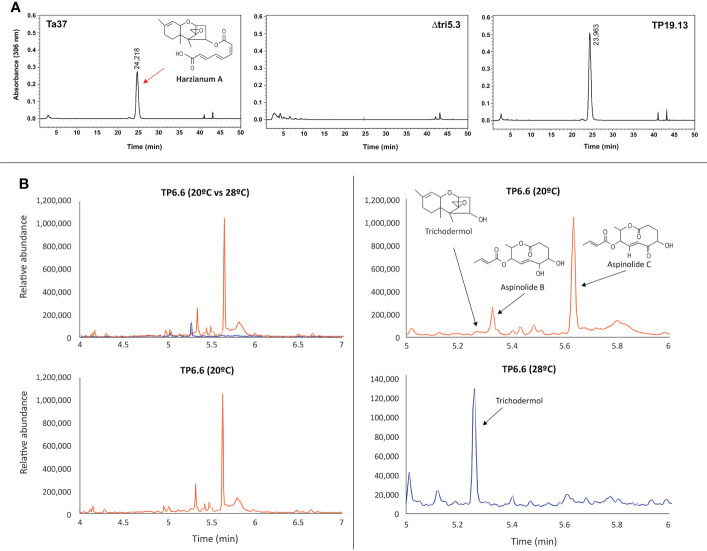
**(A)** Chromatograms from HPLC-UV analysis for determination of harzianum A production by *T. arundinaceum* IBT 40837 (Ta37) (wild-type strain); Δtri5.3, a trichothecene non-producing mutant; and TP19.13 one of the three *Trichoderma* sp. strains recovered in the present work and selected for further studies. Red arrow indicates the peak corresponding to HA. Ta37 and Δtri5.3 strains have been included for comparative purposes. **(B)** Chromatograms from GC-MS analysis for production of trichothecene intermediates (trichodermol) and aspinolides A and B from 7 days old culture supernatants of TP6.6 strain. Chromatograms in blue correspond to metabolites detected in cultures incubated at 28°C, and those in orange correspond to those incubated at 20°C. Note that a different scale was used in the lower right panel.

HA production by isolates TP6.6, TP15.11 and TP19.13 was quantified in 48-h PDB cultures. The levels of HA produced were 46.0 μg/mL, 145.27 μg/mL, and 488.54 μg/mL, respectively. The previously well-characterized *T. arundinaceum* strain IBT 40837 (Ta37), which was used as a positive control, produced HA at 250.32 µg/mL (**Figure 3A**).

#### 3.3.2 Analysis of strain TP6.6 by GC-MS to detect trichothecene biosynthetic intermediates and other secondary metabolites

Because isolate TP6.6 produced the lowest levels of HA of the three isolates examined, it was selected for further analysis by GC-MS to determine whether it produced other secondary metabolites. For this analysis, TP6.6 was grown for 7 days in YEPD medium at 20 or 28°C. When TP6.6 was grown at 28°C, it produced significant levels of trichodermol, a C4-hydroxylated precursor of HA. When grown at 20°C, TP6.6 produced a much lower level of trichodermol but higher levels of the polyketides aspinolides B and C (**Figure 3B**). In previous studies of *T. arundinaceum*, aspinolide production increased when HA production was blocked by inactivation of trichothecene biosynthetic genes or when conditions for HA production were not optimal (Malmierca et al., 2015; Izquierdo-Bueno et al., 2018; Cardoza et al., 2019; 2022).

### 3.4 Antifungal activity of the *Trichoderma* spp. isolated in the current study

The membrane and direct confrontation assays were both used to assess antifungal activity of bean-field isolates TP6.6 and TP19.13 against the phytopathogens *R. solani* and *S. sclerotiorum*. These pathogens were selected because they cause some of the most important diseases, e.g., root rots, affecting bean yield in the province of León. The previously characterized wild-type (IBT 40837) and trichothecene-nonproducing Δtri5.3 (=ΔT5.3) strains of *T. arundinaceum* were used as controls in the assays. Mutant strain ΔT5.3 was used to determine the importance of trichothecene production on antifungal activity of the strains used in this study.

**Figure 4:**
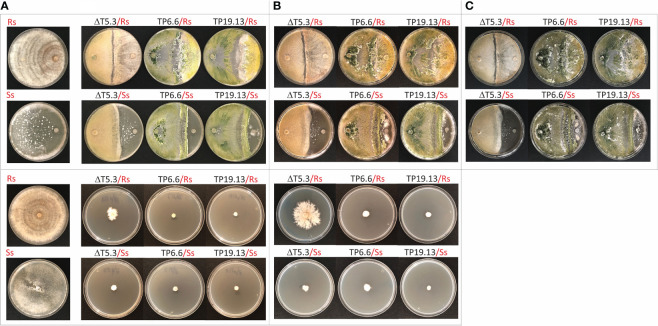
Upper panels. Direct confrontation assays of *Trichoderma* strains, Δtri5.3 (= ΔT5.3), TP6.6 and TP19.13, versus the pathogens *R. solani* R43 (Rs) (first row), or *S. sclerotiorum* S47 (Ss) (second row). Plates were incubated for one **(A)**, two **(B)**, or three weeks **(C)**. Control plates with the pathogens without *Trichoderma* are included in the left column. In all confrontations pathogen´s plugs are located at the right and *Trichoderma* plugs are located at the left of the plates. Lower panels. Cellophane membrane assays to analyze the antifungal activity of the same *Trichoderma* strains indicated above against Rs (third row) and Ss (forth row). Plates were incubated for one **(A)** or two **(B)** weeks after placement of the pathogen plug. Control plates with the pathogens grown without *Trichoderma* metabolites are included at the left column. Note that control plates were incubated in the same conditions as the plates where the direct confrontation or the cellophane membrane assays were performed.

In the confrontation assay, isolates TP6.6 and TP19.13 had overgrown *R. solani* after one week of incubation. After two and three weeks, TP6.6 and TP19.13 had completely parasitized *R. solani*, as we evident by *R. solani* no longer being visible. In contrast, the trichothecene-nonproducing mutant, Δtri5.3, had not overgrown *R. solani* even after three weeks (**Figure 4**). Also in the confrontation assay, TP6.6 and TP19.13 overgrew *S. sclerotiorum* but did so more slowly than with *R. solani*. That is, there was no apparent overgrowth after one week, but there was after two weeks (**Figure 4**). In addition, TP6.6 overgrew *S. sclerotiorum* slightly more rapidly than TP19.13. Δtri5.3 mutant lost this ability and did not overgrow the *S. sclerotiorum* colony after three weeks of growth.

The results of the membrane assay with *R. solani* were comparable to those of the confrontation assay in that both TP6.6 and TP19.13 almost completely inhibited the growth of *R. solani* after one and two weeks of incubation, while mutant Δtri5.3 caused markedly lower levels of inhibition. In contrast, all three *Trichoderma* strains almost completely inhibited growth of *S. sclerotiorum* at both time points (**Figure 4**).

Together, the results of the confrontation and membrane assays indicate that both bean-field isolates, TP6.6 and TP19.13, have strong antifungal activity against the two phytopathogens tested. The reduced antifungal activity of the trichothecene-nonproducing mutant, Δtri5.3, against *R. solani* in both assays indicates that HA production has an important role in the activity against *R. solani.* In contrast, the effects on growth of *S. sclerotiorum* by the trichothecene-nonproducing mutant Δtri5.3 versus trichothecene-producing bean-field isolates differed in the confrontation and membrane assays. In the confrontation assay, Δtri5.3 did not affect the growth of *S. sclerotiorum* in the same way as the bean-field isolates, but in the membrane assay, Δtri5.3 had the same inhibitory effect on the growth of *S. sclerotiorum* as the bean-files isolates, indicating that in membrane assay other metabolites produced by Δtri5.3, different from HA, are responsible for that antifungal activity against *S. sclerotiorum*.

### 3.5 Analysis of trichothecene biosynthetic loci in bean-field isolates

**Figure 5:**
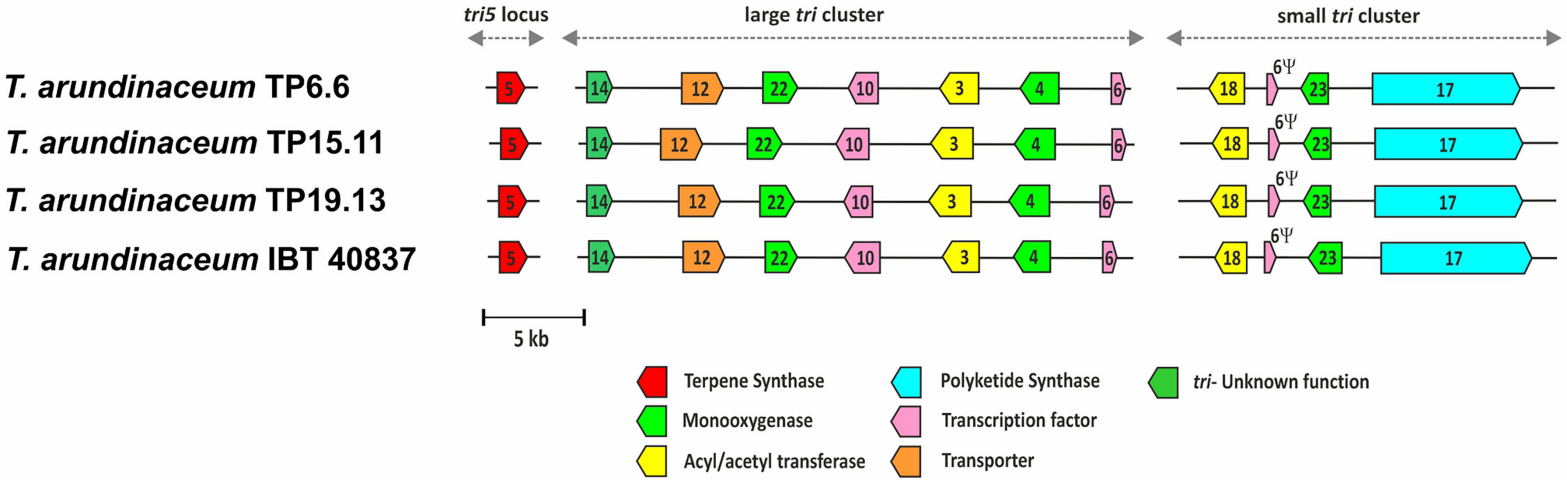
Trichothecene biosynthetic genes (*tri*) in the genomes of the three *Trichoderma* strains isolated in the current study. *Trichoderma arundinaceum* IBT40837 was included for comparative purposes. Numbers inside arrows indicate the *tri* gene illustrated, e.g., 5 corresponds to *tri5* gene. Different arrow colors indicate different gene functions as shown at the bottom of the figure. Ψ corresponds to a *tri6*-pseudogene.

Genome sequences were also used to examine the trichothecene biosynthetic loci. A summary of the main features found in the genome sequences of the bean-field isolates TP6.6, TP15.11 and TP19.13 are shown in **Table 1**. Analysis of the sequences indicated that the three isolates had the three trichothecene biosynthetic loci previously described in the reference *T. arundinaceum* strain IBT 40837. The three loci are the *tri5* locus, large *tri* cluster, and the small *tri* cluster (**Figure 5**). The order and orientation of genes within the loci were the same in the three bean-field isolates and strain IBT 40837. In addition, a heretofore unrecognized pseudogenized copy of the trichothecene regulatory gene *tri6* was found in the small *tri* cluster locus. Reanalysis of previously analyzed genome sequences indicated that a *tri6* pseudogene (=6Ψ) was present at at the genomic location in other HA-producing *Trichoderma* species (e.g., *T. turrialbense* and *T. protrudens*) (data not shown).

**Table 1:** Summary of genome sequence assembly statistics for the three strains of *T. arundinaceum* isolated during the current study.

Sample id	#contigs	Largest contig (bp)	Total length (bp)	cds*	%GC	N50
**TP6.6**	196	4,913,079	38,630,653	11,426	47.42	1,552,653
**TP15.11**	189	3,058,384	37,857,240	11,396	48.94	1,690,046
**TP19.13**	167	3,897,532	37,843,060	11,307	47.91	1,860,499

*Number of coding sequences (cds) deduced from each genome sequence through Augustus analysis.

### 3.6 *tri5* phylogenetic analysis

**Figure 6:**
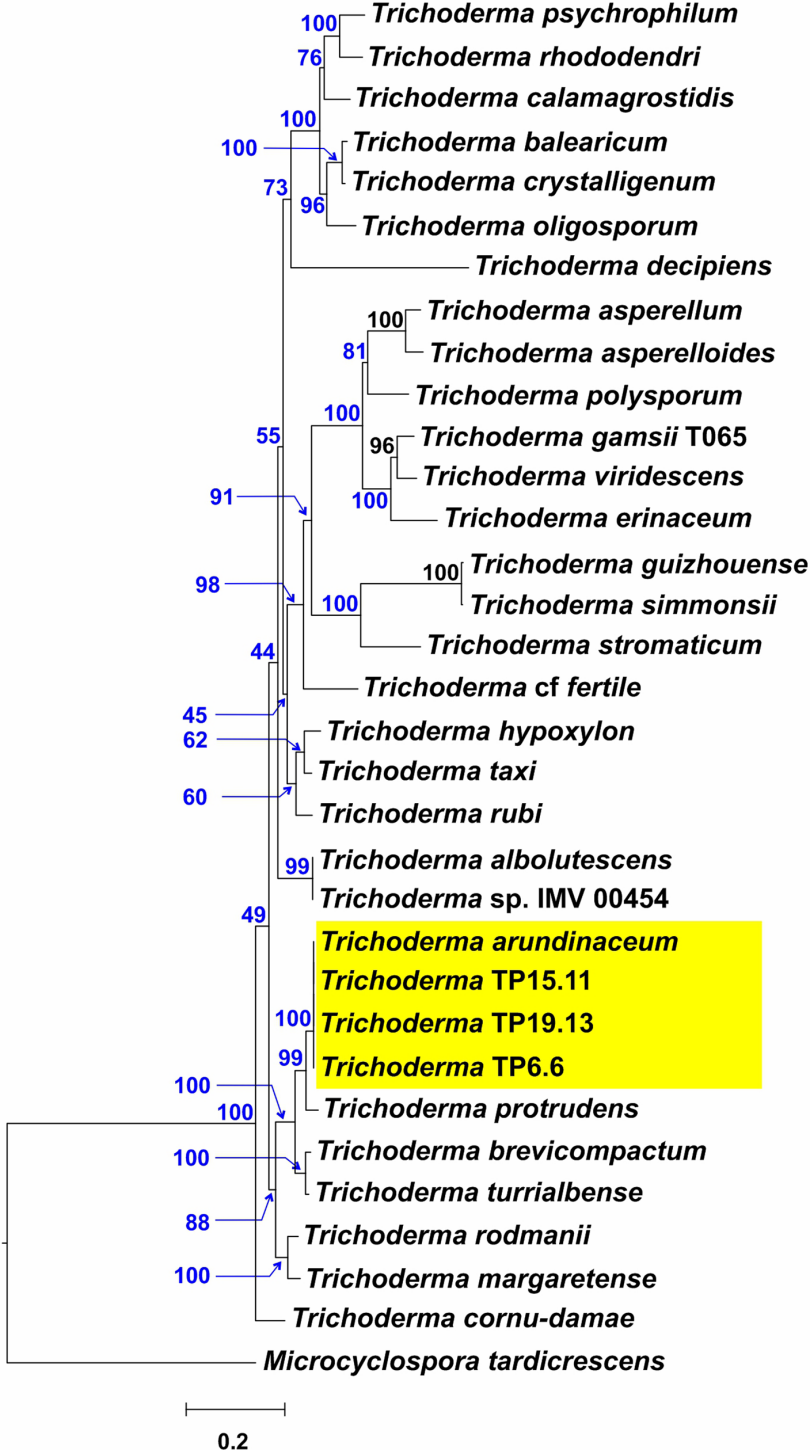
Phylogenetic tree inferred by maximum likelihood analysis of full-length exon sequences of *tri5* from TP6.6, TP15.11 and TP19.13 strains, and other 30 *Trichoderma* species. Numbers on each branch (blue type) are bootstrap values based on 1000 pseudoreplicates. Yellow rectangle points to the *tri5* from the three isolates selected in the current study.

An ML analysis was performed to assess the phylogenetic relationships of *tri5* homologs from the three bean-field isolates to homologs from 29 *Trichoderma* species (**Table S2**) (Gutiérrez et al., 2021). In the resulting ML tree, the *tri5* orthologs from TP6.6, TP15.11 and TP19.13 formed an exclusive and well-supported clade (bootstrap value 100%) with the ortholog from the *T. arundinaceum* reference strain IBT 40837 (**Figure 6**), providing further evidence for the close relationship of the bean-field isolates and the reference strain.

### 3.7 Microbiome analysis

**Figure 7:**
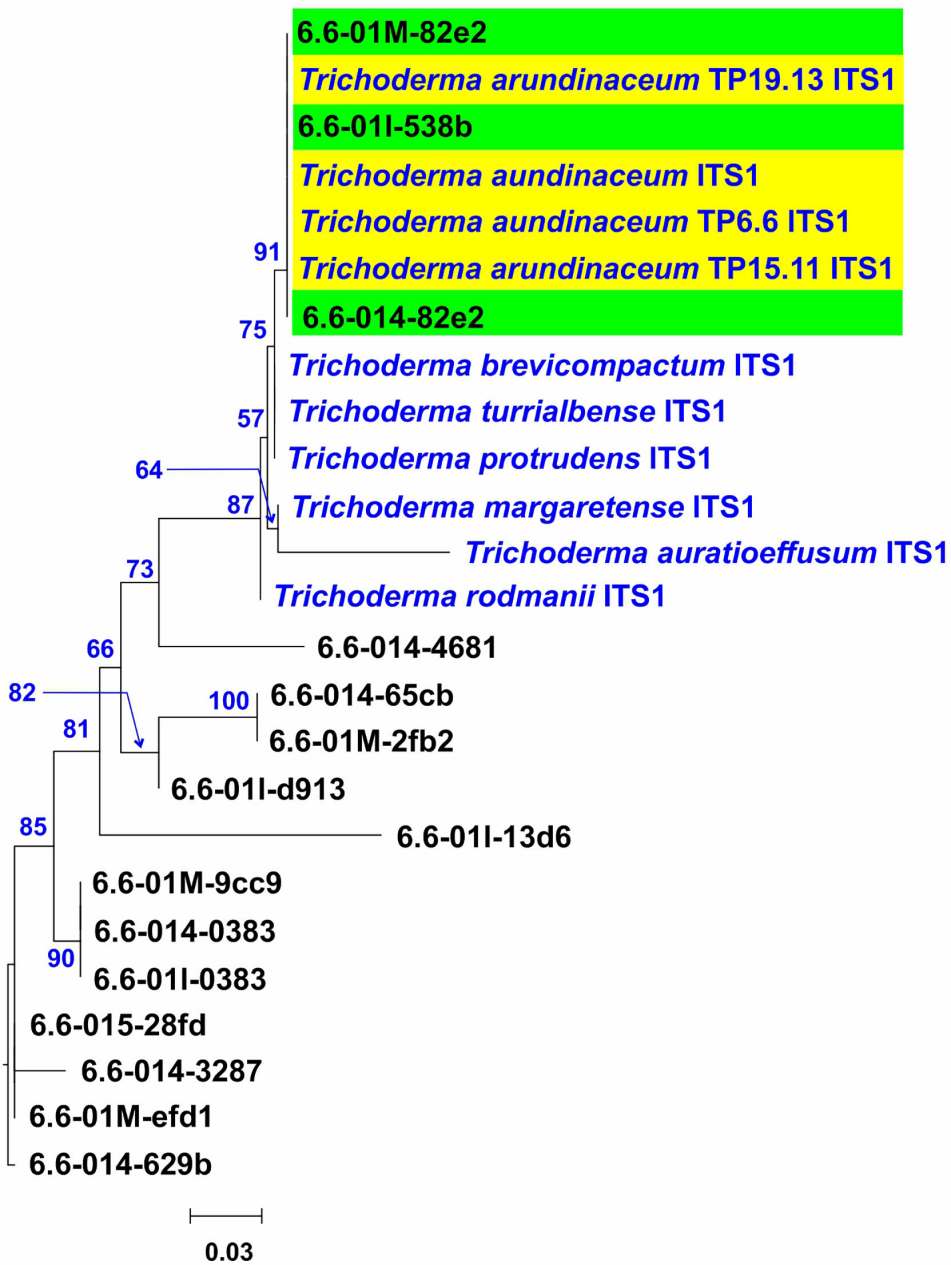
Phylogenetic tree inferred by maximum likelihood analysis of selected ITS1 sequence (193 bp) from amplicons retrieved from the microbiome analysis (black type, 6.6-01#) and *Trichoderma* species in the Brevicompactum clade (blue type sequences), The amplicon sequences selected for this analysis were those with the highest scores in BLASTn analysis in which the ITS1 sequence from the *T. arundinaceum* reference strain IBT 40837 was queried against all ITS1 amplicon sequences derived from four soil samples collected from Plot 6. Numbers near each branch (blue type) are bootstrap values based on 1,000 pseudoreplicates. Yellow rectangle denotes sequences of the three *T. arundinaceum* strains isolated in the current study and the reference strain IBT 40837. Green rectangles denote amplicon sequences that are identical to ITS1 from reference strain IBT 40837.

A DNA-sequencing-based microbiome approach was used to determine if the *Trichoderma* trichothecene-producer strains isolated following traditional microbiological procedures can be also detected following that strategy. For this analysis, DNA libraries were prepared from four soil samples collected from plot number 6, the same location from which *T. arundinaceum* isolate TP6.6 was recovered. From these four libraries, a total of 1,944 fungal ITS1 sequences were generated and then used as an in-house database for a BLASTn analysis with the ITS1 sequence from *T. arundinaceum* IBT 40837 serving as the query. The ITS1 sequences showing the highest hit to the IBT 40837 sequence were then used as queries in BLASTn analysis with another in-house genome sequence database, which included genome sequences from all species in the *Brevicompactum, Psychrophila* and *Rubi* clades and from the species *T. albolutescens* (i.e., all trichothecene-producing species of *Trichoderma*). This analysis identified three ITS1 sequences, 6.6-01M-82e2, 6.6-014-82e2, and 6.6-01I-538b, which were most closely related to ITS1 sequences from members of clade *Brevicompactum*. An ML tree was then inferred from an alignment of 6.6-01M-82e2, 6.6-014-82e2, and 6.6-01I-538b, ITS1 sequences from isolates TP6.6, TP15.11, and TP19.13, all publicly available ITS1 sequences from species in clade *Brevicompactum*, and 12 additional ITS1 sequences from our microbiome analysis that were similar but not identical to ITS1 sequences from members of clade *Brevicompactum*. In the resulting tree, sequences 6.6-01M-82e2, 6.6-014-82e2, and 6.6-01I-538b formed an exclusive and well-supported clade (bootstrap value 91) with the ITS1 sequences from *T. arundinaceum* IBT 40837 and the bean-field isolates TP6.6, TP15.11 and TP19.13 (**Figure 7**). These data indicated that *T. arundinaceum* strains previously selected from soils by classical microbiological methods were also detected by microbiomic approaches.

### 3.8 Detection of biosynthetic gene clusters (BGC) putatively involved in the synthesis of secondary metabolites from the genome sequences of strains TP6.6, TP15.11 and TP19.13

Genome sequence data from bean-field isolates TP6.6, TP15.11 and TP19.13 were subjected to antiSMASH analysis to identify the secondary metabolite biosynthetic gene clusters (BGCs) present in the three genomes (Medema et al., 2011). The results of the analysis indicated that the number of BGCs as well as the numbers of PKS, NRPS, Hybrid PKS-NRPS and terpene synthase genes in the three bean-field isolates were similar to those in the reference *T. arundinaceum* strains IBT 40837, which is predicted to have 50 BGCs; 23 PKS genes, 25 NRPS genes, 5 hybrid PKS-NRPS genes, and 10 terpene synthase genes (**Table 2**).

**Table 2:** Number of biosynthetic gene clusters (BGC), PKS, NRPS, hybrid PKS-NRPS, and terpene synthase encoding genes found in the genomes of the three isolates selected in the current study, as deduced from Augustus and antiSMASH analyses.

Sample id	#BGC	PKS*	NRPS**	Hybrid PKS-NRPS	Terpene synthases
**TP6.6**	51	25	27	5	10
**TP15.11**	50	25	26	5	9
**TP19.13**	51	25	25	5	10
Ta37***	50	23	25	5	10

*PKS, Polyketide synthase.

**NRPS, Non-ribosomal peptide synthetase.

***Data from strain Ta37 (=*Trichoderma arundinaceum* IBT40837) are included only for comparative purposes, and were retrieved from data previously reported by our group (Lindo et al., 2018; Proctor et al., 2018).

### 3.9 Effect of HA production on the germination and growth of bean plants in the presence of the pathogens *R. solani* and *S. sclerotiorum*

**Figure 8:**
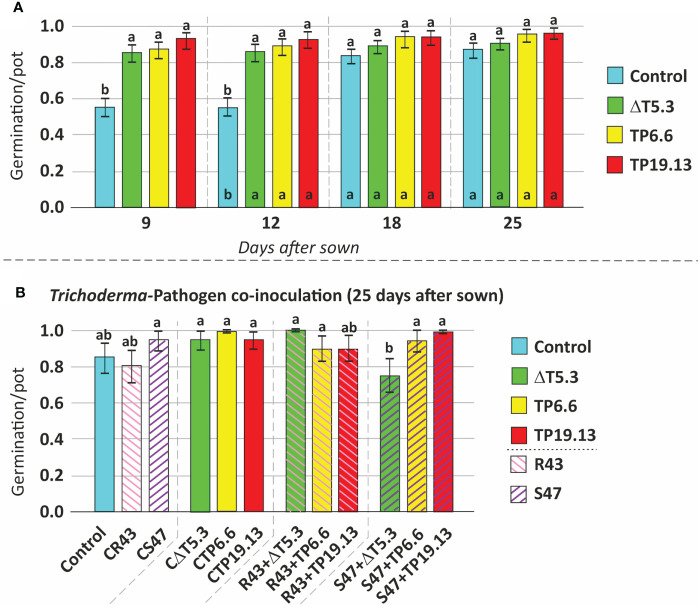
Mean comparison (Duncan’s multiple range test) for: **(A)** bean seed germination without any *Trichoderma* treatment (Control) and coated with the three *Trichoderma* strains used in this study (ΔT5.3, TP6.6, and TP19.13), incubated for 9 to 25 days after sowing, and **(B)** germination of bean seeds after 25 days from sown, using as source of variation the Pathogen [*R. solani* R43 (=R43) and *S. sclerotiorum* (=S47)], without any *Trichoderma* treatment (control) and coated with the three *Trichoderma* strains used in this study (ΔT5.3, TP6.6, and TP19.13). CR43 and CS47 correspond to seeds grown in substrate infected with *R. solani* R43 and *S. sclerotiorum* S47, respectively. CΔT5.3, CTP6.6, and CTP19.13= seeds inoculated with spores of ΔT5.3, TP6.6, and TP19.13, respectively, which were grown in substrate without previous pathogen inoculation. R43+# and S47+#= seeds coated with the *Trichoderma* strain indicated on each name (#=ΔT5.3, TP6.6, or TP19.13) and grown in a substrate previously infected with *R. solani* R43 and *S. sclerotiorum* S47, respectively. Different lowercase letters (a, b) above the bars indicate statistically significant differences between the analyzed treatments (p<0.05). ΔT5.3= Δtri5.3.

**Figure 9:**
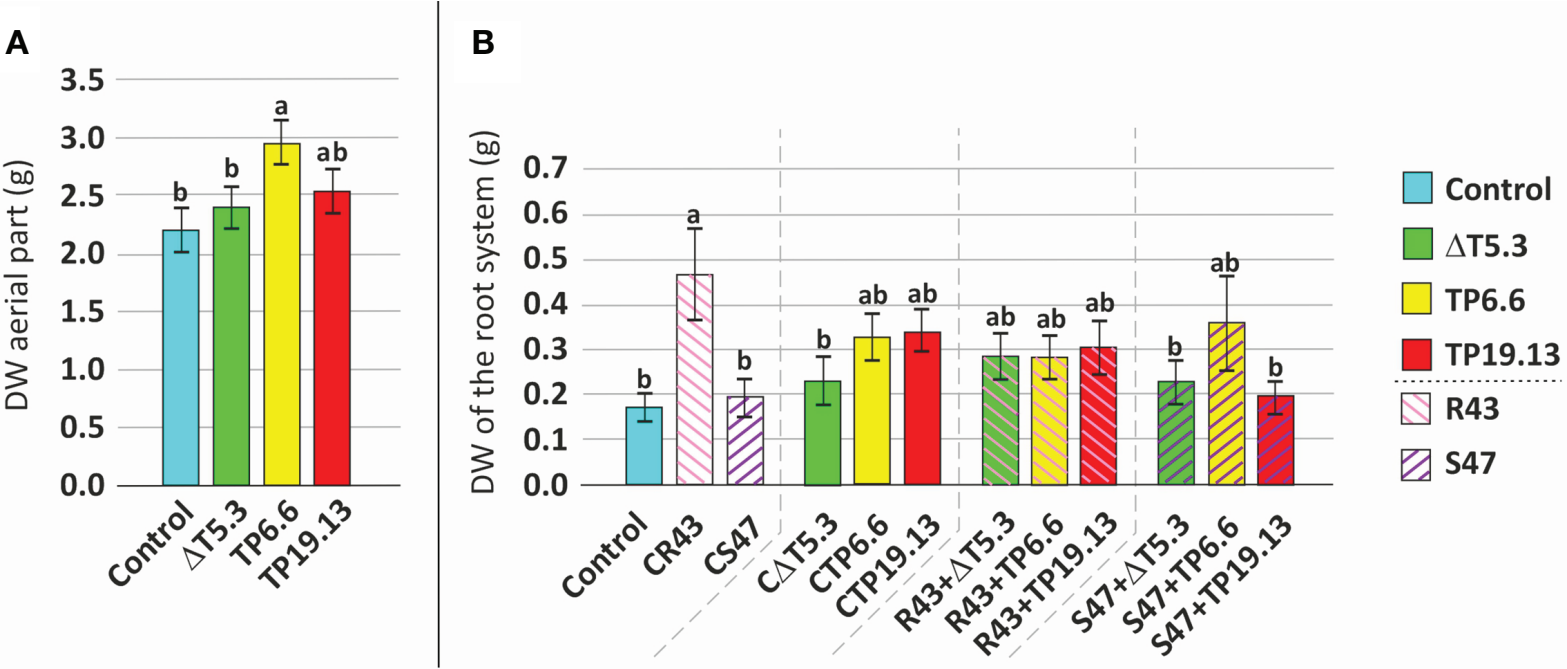
Mean comparison (Duncan’s multiple range test) of dry weight (DW) of 45 days old bean plants. **(A)** Evaluation of DW of aerial parts from plants grown from seeds without any *Trichoderma* treatment (Control) and coated with the three *Trichoderma* strains used in this study (ΔT5.3, TP6.6, and TP19.13). **(B)** Analysis of DW of root system using as source of variation the pathogen x *Trichoderma* interaction. Within **(A, B)**, different letters above bars indicate significant differences (p<0.05). For the description of the different treatments (x-axis) see legend to **Figure 8**. ΔT5.3= Δtri5.3.

Isolates TP6.6 and TP19.13 were selected for this analysis because they produced the lowest and the highest, respectively, levels of HA among the three *T. arundinaceum* bean-field isolates recovered in the study. Bean seeds from the “Riñón” variety were coated with spores of *T. arundinaceum* TP6.6 or TP19.13 and then sown in pots containing soil infested with *R. solani* and *S. sclerotiorum*. The next parameters were analyzed: **1. Germination**- In the soil that was not infested with the plant pathogens, bean seeds coated with either *T. arundinaceum* isolate had a higher germination rate than the uncoated control seeds only at 9 and 12 days after sowing but not at 18 or 25 days (**Figure 8A**; **Table S3**). In contrast, in soils that were infested with the plant pathogens, differences between the presence of pathogen and *Trichoderma* were only statistically significant for conditions where *S. sclerotiorum* was present in the substrate at 25 days after sowing. In this condition a lower germination rate was observed for seeds inoculated with Δtri5.3 and grown in substrate amended with this pathogen, compared to the control with only the pathogen and also to the seeds inoculated with the TP6.6 or TP19.13 (HA-producers) grown in the same conditions, i.e., in substrate amended with S47 (**Figure 8B**; **Table S3**). These results were not observed when the pathogen *R. solani* was used to perform the same analyses (**Figure 8B**). Finally, the presence of the pathogens without *Trichoderma* did not significantly affect germination (data not shown). **2. Growth**- Plant growth was assessed using separate dry weight measurements of aerial plant parts and the root system. Growth of aerial plant parts was significantly greater in the TP6.6-inoculation treatment compared to Δtri5.3-inoculation treatments (**Figure 9A**; **Table S4**). Furthermore, root system growth was higher in plants grown in *R. solani*-infested soil compared to uninfested soil. However, this increased root growth in *R. solani*-infested soil was reduced by the presence of any of the *T. arundinaceum* isolates analyzed in this work. Root system growth was not affected in soil infested with *S. sclerotiorum* alone. However, the growth was increased in the *S. sclerotiorum*-TP6.6 co-inoculation treatment compared to the *S. sclerotiorum*-alone, *S. sclerotiorum*-TP19.13 co-inoculated, or *S. sclerotiorum*-Δtri5.3 treatments, but these differences were not statistically significant (**Figure 9B**; **Table S4**; **Figure S2**).

### 3.10 Effect of trichothecene producing *Trichoderma* strains on the bean transcriptome

Leaves detached from plants under the different treatments were used in the present study and processed as described in the *Material and Methods* section. A total of 1.238,9 million reads were obtained, after trimming and processing the raw reads (**Table S5**). A differentially expressed gene (DEG) analysis was performed to determine the differential expression of bean genes in the conditions analyzed. In this analysis, even though leaves from plants treated with the three mentioned strains were used for mRNA isolation, only TP19.13 and Δtri5.3 strains were analyzed by RNAseq, based on the higher level of production of HA shown by TP19.13 compared to TP6.6. However, mRNA from plants treated with the strain TP6.6 were further included in the qPCR analysis, together with those from plants treated with TP19.13 and Δtri5.3, in order to validate the RNAseq results.

Data of the whole transcriptome analysis, including the 9 comparisons analyzed in the present work, indicated that among the 32,720 transcripts detected in the *Phaseolus vulgaris* genome (assembly PhaVulg1_0) (https://ftp.ncbi.nlm.nih.gov/genomes/all/GCF/000/499/845/GCF_000499845.1_PhaVulg1_0/), 14,942 were differentially expressed (**Table S6**; **Figure S3**). However, our analysis focused on defense-related genes and also on genes related to hormone biosynthesis, and to hormone signaling pathways, which could be also related to plant defense response. To carry out this study, 15 genes were initially selected: *CHS.3* (involved in phenylpropanoid biosynthesis); *CPRD14.4* and *CPRD14.3* (stress response); *Lox2.3* and *Lox1.3* (biosynthesis of antimicrobials and oxylipins- important as defense signaling molecules); *AOS.4* (allene oxide synthase- oxylipins pathway); *LTP2.1, MMP2.2, TSI-1.2, PR1.2* and *PR16a* (pathogenesis related genes); *GTSa.1* (oxidative stress); *Ch5b.3* (endochitinase precursor- pathogenesis related gene); *ERF5* (ethylene-responsive transcription factor 5- ethylene signaling pathway); and *SIP.3* (Syringolide induced protein B13.1.9- defense response) (**Table S7**).

Data from RNAseq analysis were later validated by qPCR, using custom primer pairs designed for each of the analyzed genes, whose amplification efficiencies range between 85.4% and 114% (**Table S8**). This analysis also included the data from TP6.6 strain, which was not used in the RNAseq analysis. The actin gene was used as a housekeeping internal control (=reference gene), and bean plants not inoculated with *Trichoderma* as the reference sample.

**Figure 10:**
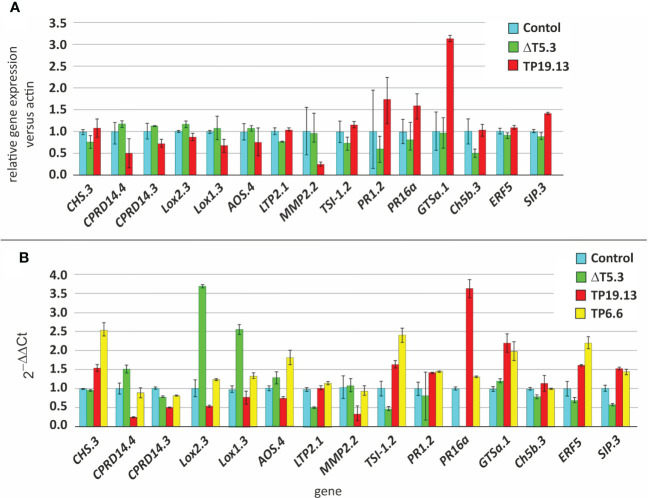
**(A)** Relative expression level of 15 defense-related genes selected for the present study as deduced from the RNAseq analysis. Relative expression was inferred as the ratio of the normalized expression level (TMM) of each gene versus the actin. The values were calculated as the average and standard deviation of two biological replicates. **(B)** qPCR analysis to determine relative expression of the same 15 defense-related genes as above. Arithmetic average and standard deviation data from three technical replicates were represented. Analysis was performed by the 2^-ΔΔCT^ method (Livak and Schmittgen, 2001), using the actin gene as a housekeeping internal control, and bean plants not inoculated with *Trichoderma* as the reference sample.

Expression of many of the analyzed defense-related genes was affected by the production of HA, since the expression of these genes was significantly different in plants inoculated with the trichothecene producing strains, TP6.6 and TP19.13, in comparison to the trichothecene non-producing mutant Δtri5.3 (**Figure 10**). In addition, when the qPCR results were compared to those from the RNAseq analysis, a correlation in the tendencies of both groups of data was observed (**Figure 10**), even though the absolute values were different, which should be expected due to great methodology differences in the obtention of the data between those procedures. Thus, combining results from both analyses (qPCR and RNAseq), among the 15 genes analyzed by qPCR, only *Lox2.3* and *Lox1.3* (biosynthesis of oxylipins) were significantly downregulated in plants inoculated with the two HA producers used in this analysis (TP19.13 and TP6.6) versus plants inoculated with the HA-non producing mutant (ΔT5.3); and three genes (*LPT2.1, PR16a*-pathogenesis related-, and *SIP3*- related to defense response-) were significantly upregulated in the same comparison as above (**Figure 10**).

We also analyzed the RNAseq data for differential expression of other defense-related genes, including genes encoding for enzymes involved in the biosynthesis of ethylene, abscisic acid (ABA), phytoalexins, genes related with oxidative stress [reactive oxygen species (ROS), H_2_O_2_, glutathione], genes related with cell wall biosynthesis, pathogenesis related (PR), and related to alternative secondary metabolite biosynthetic genes. In this analysis, genes showing expression ratios >=2 or <=0.5, with p values <=0.05 between the two compared samples were considered.

**Figure 11:**
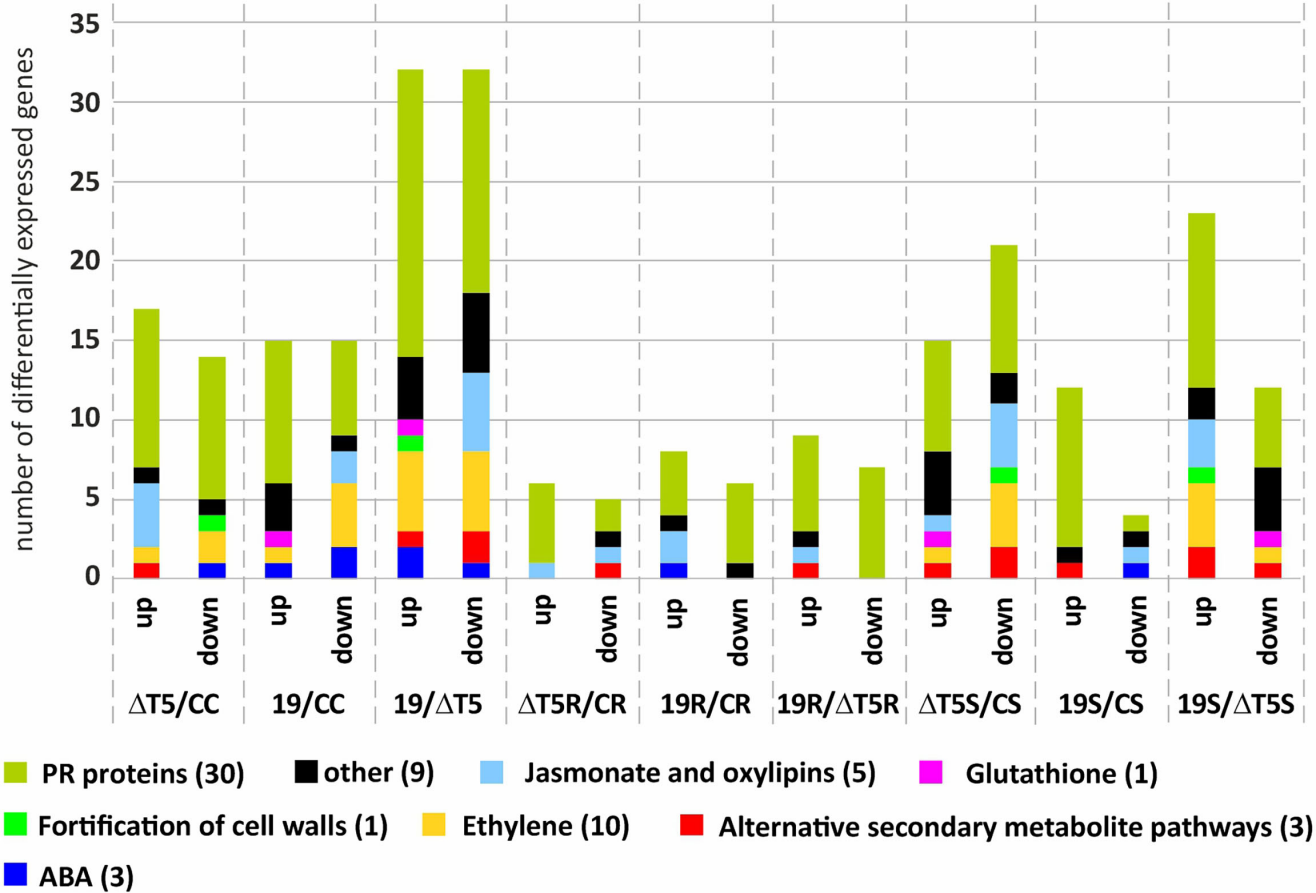
Representation of the number of genes up- or down-regulated within the different categories analyzed (upregulated: >=2, downregulated: <=-2). Comparisons represented are as follows: ΔT5/CC (plants inoculated with the strain ΔT5.3 versus non-inoculated plants), 19/CC (plants inoculated with the strain TP19.13 versus non-inoculated plants), 19/ΔT5 (plants inoculated with TP19.13 versus plants inoculated with ΔT5.3), 19R/CR (plants inoculated with T19.13 versus non-inoculated plants, both grown in the substrate infected with the pathogen *R. solani* R43), 19R/ΔT5R (plants inoculated with T19.13 versus plants inoculated with ΔT5.3, both grown in substrate infected with *R. solani* R43), ΔT5S/CS (plants inoculated with ΔT5.3 versus non-inoculated plants, both grown in substrate infected with *S. sclerotiorum* S47), 19S/CS (plants inoculated with TP19.13 versus non-inoculated plants, both grown in substrate infected with *S. sclerotiorum* S47), and 19S/ΔT5S (plants inoculated with TP19.13 versus plants inoculated with ΔT5.3, both grown in substrates amended with *S. sclerotiorum* S47). Sixty-four genes were selected for this analysis (**Table 3**). The genes were grouped based on to their blast annotated function as indicated at the bottom part of the figure. ΔT5= ΔT5.3= Δtri5.3.

As result, the comparison with the greatest differences in expression was TP19.13 vs. Δtri5.3; 31 genes were upregulated and 31 were downregulated (**Figure 11**). This indicates that production of HA by TP19.13 had a marked effect on the plant defense response. Furthermore, in all conditions that included *R. solani*-infested soil, the number of differentially expressed genes was lower than in the treatments with uninfested soil or *S. sclerotiorum*-infested soil. Regarding the differences observed between the different groups of genes, in the absence of a plant pathogen, inoculation with Δtri5.3 resulted in upregulation of genes related with jasmonate, oxylipins, and ethylene signaling pathways versus inoculation with TP19.13. However, the opposite was observed in treatments with *S. sclerotiorum* (**Figure 11**).

Finally, regarding the individual genes analyzed by RNAseq, four chitinases-encoding genes were up-regulated by TP19.13 versus Δtri5.3 (gene codes in **Table 3**: PHAVU_009G116300g, _009G1165001g, _003G158500g, and _001G046400g), and only one chitinase was upregulated at a higher level by Δtri5.3 versus TP19.13 (gene code in **Table 3**: PHAVU_011G167000g). The expression pattern in the ethylene-related genes was variable, with some genes upregulated in plants inoculated with Δtri5.3, and others upregulated in plants inoculated with TP19.13.

**Table 3:** Defense-related genes analyzed from the RNA-seq data.

Gene code	Transcript code	Defense related gene group	Annotated function
PHAVU_003G004800g	XM_007153015.1	ABA*	abscisic acid 8’-hydroxylase 2
PHAVU_009G077200g	XM_007136760.1	ABA	abscisic acid receptor PYL2
PHAVU_003G292200g	XM_007156451.1	ABA	abscisic acid 8’-hydroxylase 4
PHAVU_009G192400g	XM_007138186.1	alternative SM pathways	flavonoid 3’-monooxygenase
PHAVU_002G047500g	XM_007157097.1	alternative SM pathways	2-hydroxyisoflavanone dehydratase
PHAVU_008G287200g	XM_007142453.1	alternative SM pathways	NAD(P)H-dependent 6’-deoxychalcone synthase
PHAVU_003G020000g	XM_007153196.1	Ethylene	1-aminocyclopropane-1-carboxylate oxidase homolog 4-like
PHAVU_003G020100g	XM_007153197.1	Ethylene	1-aminocyclopropane-1-carboxylate oxidase homolog 4-like
PHAVU_005G058800g	XM_007149239.1	Ethylene	1-aminocyclopropane-1-carboxylate oxidase homolog 1
PHAVU_004G069900g	XM_007151656.1	Ethylene	ethylene-responsive transcription factor ERF113-like
PHAVU_006G106100g	XM_007147161.1	Ethylene	ethylene-responsive transcription factor 1B-like
PHAVU_002G055800g	XM_007157198.1	Ethylene	Ethylene-responsive transcription factor 5
PHAVU_002G315900g	XM_007160308.1	Ethylene	ethylene-responsive transcription factor ERF117-like
PHAVU_003G020200g	XM_007153198.1	Ethylene	1-aminocyclopropane-1-carboxylate oxidase homolog 4-like
PHAVU_009G129500g	XM_007137408.1	Ethylene	1-aminocyclopropane-1-carboxylate oxidase 5
PHAVU_002G149500g	XM_007158332.1	Ethylene	Ethylene-responsive transcription factor ESR2
PHAVU_002G031700g	XM_007156897.1	Cell wall fortification	laccase-11
PHAVU_005G054100g	XM_007149185.1	Glutathione	putative glutathione S-transferase
PHAVU_010G134800g	XM_007135443.1	Jasmonate and oxylipins	linoleate 9S-lipoxygenase 1
PHAVU_010G134900g	XM_007135444.1	Jasmonate and oxylipins	linoleate 9S-lipoxygenase 1
PHAVU_005G156900g	XM_007150427.1	Jasmonate and oxylipins	Linoleate 9S-lipoxygenase-4
PHAVU_010G135000g	XM_007135445.1	Jasmonate and oxylipins	Linoleate 9S-lipoxygenase 1
PHAVU_001G012300g	XM_007160668.1	Jasmonate and oxylipins	jasmonate O-methyltransferase
PHAVU_001G039900g	XM_007161004.1	Other	putative WRKY transcription factor 50
PHAVU_001G042200g	XM_007161033.1	Other	putative WRKY transcription factor 40
PHAVU_007G209000g	XM_007145029.1	Other	putative WRKY transcription factor 47 isoform X1
PHAVU_L005700g	XM_007163856.1	Other	cinnamoyl-CoA reductase 1-like
PHAVU_008G103500g	XM_007140270.1	Other	GDSL esterase/lipase CPRD49-like
PHAVU_005G034700g	XM_007148969.1	Other	cinnamoyl-CoA reductase 1-like
PHAVU_001G194900g	XM_007162900.1	Other	NDR1/HIN1-like protein 10
PHAVU_001G088200g	XM_007161602.1	Other	putative WRKY transcription factor 75
PHAVU_010G111900g	XM_007135170.1	Other	putative WRKY transcription factor 46
PHAVU_006G078400g	XM_007146820.1	PR protein	non-specific lipid-transfer protein 1-like
PHAVU_006G197300g	XM_007148243.1	PR protein	pathogenesis-related protein 1-like
PHAVU_011G167000g	XM_007133223.1	PR protein**	acidic endochitinase-like
PHAVU_009G116300g	XM_007137243.1	PR protein	class I chitinase
PHAVU_009G1165001g	XM_007137245.1	PR protein	class I chitinase
PHAVU_001G046400g	XM_007161085.1	PR protein	acidic mammalian chitinase
PHAVU_003G158500g	XM_007154854.1	PR protein	class V chitinase-like
PHAVU_011G065300g	XM_007132018.1	PR protein	osmotin-like protein
PHAVU_004G123100g	XM_007152295.1	PR protein	peroxidase 66
PHAVU_002G084500g	XM_007157554.1	PR protein	ribonuclease 3
PHAVU_010G066500g	XM_007134613.1	PR protein	Non-specific lipid-transfer protein 1
PHAVU_006G197100g	XM_007148241.1	PR protein	pathogenesis-related protein 1-like
PHAVU_008G086800g	XM_007140072.1	PR protein	cationic peroxidase 1
PHAVU_008G086900g	XM_007140073.1	PR protein	cationic peroxidase 1
PHAVU_009G232000g	XM_007138661.1	PR protein	pathogen-associated molecular patterns-induced protein A70-like
PHAVU_004G021000g	XM_007151077.1	PR protein	metalloendoproteinase 1-like
PHAVU_002G201700g	XM_007158959.1	PR protein	putative polygalacturonase-inhibiting protein precursor
PHAVU_004G002700g	XM_007150827.1	PR protein	Non-specific lipid-transfer protein 2
PHAVU_009G252300g	XM_007138892.1	PR protein	pathogenesis-related protein STH-2-like
PHAVU_002G201600g	XM_007158958.1	PR protein	putative polygalacturonase-inhibiting protein precursor
PHAVU_005G071400g	XM_007149387.1	PR protein	defensin-like protein
PHAVU_008G129500g	XM_007140581.1	PR protein	endoribonuclease Dicer homolog 2-like isoform X1
PHAVU_003G109600g	XM_007154269.1	PR protein	pathogenesis-related protein 2-like
PHAVU_008G192300g	XM_007141339.1	PR protein	thaumatin-like protein
PHAVU_003G109500g	XM_007154268.1	PR protein	pathogenesis-related protein 2-like
PHAVU_001G011300g	XM_007160657.1	PR protein	Peroxidase 9
PHAVU_003G109800g	XM_007154271.1	PR protein	pathogenesis-related protein 2-like
PHAVU_008G171400g	XM_007141084.1	PR protein	putative glucan 1,3-beta-glucosidase A
PHAVU_010G129900g	XM_007135380.1	PR protein	germin-like protein subfamily 3 member 1
PHAVU_003G1099001g	XM_007154272.1	PR protein	pathogenesis-related protein 2-like
PHAVU_006G130000g	XM_007147440.1	PR protein	peroxidase A2-like
PHAVU_002G076700g	XM_007157458.1	PR protein	metalloendoproteinase 1-like

*ABA, abscisic acid.

**PR protein, Pathogenesis Related protein.

SM, Secondary Metabolite.

## 4 Discussion

The effect of trichothecene production on plants has most often been analyzed related to plant pathogenic fungi, particularly *Fusarium* species. Some trichothecene analogs are phytotoxic and serve a critical role in virulence in certain plant-pathogen interactions (Harris et al., 1999). The first report regarding production of trichothecenes in *Trichoderma* species, describes production of HA in an unidentified *Hypocrea* strain, and correlates trichothecene with toxicity against human tumor cell lines (Lee et al., 2005). Today, trichothecene production has been reported in at least 13 *Trichoderma* species (Sun et al., 2016; Gutiérrez et al., 2021). These species have been isolated from diverse substrates, ranging from plant endophytes to soils from multiple countries and environments (Nielsen et al., 2005; Degenkolb et al., 2008; Jaklitsch, 2011; Jaklitsch and Voglmayr, 2015). However, to our knowledge, no attempts have been made to study the diversity of trichothecene-producing *Trichoderma* species in crop soils or their importance as indigenous microbes in the interaction with crops. Since the description of trichothecene production, *Trichoderma* species producing these compounds have been considered as strains outside the groups with biotechnological interest (Degenkolb et al., 2008). This rationale was based in the knowledge of the toxicity exhibited by most of the trichothecenes studied until now, in fact some *Trichoderma* trichothecenes, for example trichodermin, have also been described as highly toxic for plants (Tijerino et al., 2011).

However, works carried out in the last decade have revealed that some *Trichoderma* species produce trichothecenes that do not have phytotoxicity, e.g., *T. arundinaceum* and *T. albolutescens* (Ryu et al., 2017; Gutiérrez et al., 2020). In the opposite, trichothecenes produced by these species exhibit stimulatory effects of the plant defense response against diverse plant diseases (Malmierca et al., 2015; Malmierca et al., 2016; Ryu et al., 2017). Thus, this change in the paradigm considering trichothecenes as mycotoxins with pernicious effects on plant health, has driven the objectives of this work.

The three HA-producing *T. arundinaceum* strains recovered in the current study constituted 4.7% of the *Trichoderma* isolates recovered from the 28 soil samples processed. In general, the number of *Trichoderma* isolates recovered from each sample was low. Regardless, strains TP6.6, TP15.11 and TP19.13 constituted 33% (n=3), 33% (n=3) and 8% (n =12) of the total fungal isolates recovered from their respective samples. Previous studies have reported the isolation of *Trichoderma* strains from bean crops (Lopes et al., 2012; Mayo-Prieto et al., 2020a). However, these studies were not focused on the isolation of *Trichoderma* strains able to produce trichothecenes. Interestingly, the traditional isolation procedure (=culture, genome sequencing, phylogenetic analysis) and the microbiome approach both detected *T. arundinaceum* in soil from the plot number 6.

Genome sequences of the three *T. arundinaceum* isolates indicated a very high degree of similarity with the genome sequence of *T. arundinaceum* IBT 40837, which is an indigenous strain isolated in Iran. This indicates similar genetic profiles despite their so distant geographical origins. The analysis in these isolates of the genomic regions including the genes involved in trichothecene biosynthesis (=*tri* genes) revealed the presence of all genes previously described for strain IBT 40837 (Proctor et al., 2018; Proctor et al., 2020) with an almost total degree of synteny, with just minor differences in the length of the intergenic regions. Furthermore, a new feature was detected, corresponding to a *tri6* degenerated copy (6Ψ) that was detected in the intergenic region between *tri18* and *tri23*. This degenerated *tri6* was detected in all *Trichoderma* strains producing HA, e.g., *T. arundinaceum* IBT 40837, *T. protrudens* and T. *turrialbense*.

Consistent with the presence of the three *tri* loci in their genome sequences, the three selected isolates produced HA. In addition, a more detailed GC-MS based metabolomic analysis was carried out to detect intermediates of the HA biosynthesis, and other secondary metabolites. Data revealed production of trichodermol, an intermediate in the biosynthesis of HA that was previously reported in cultures of the reference strain *T. arundinaceum* IBT 40837 (Lindo et al., 2018). In addition, aspinolides B and C were found in *T. arundinaceum* TP6.6, but only when grown at 20°C. Production of aspinolides by *T. arundinaceum* was already reported (Malmierca et al., 2015; Izquierdo-Bueno et al., 2018; Cardoza et al., 2022), and their production together with other additional aspinolides was observed mainly when production of HA was partial or totally blocked (Malmierca et al., 2015; Izquierdo-Bueno et al., 2018). In addition, data observed in the present article also support the hypothesis that production of aspinolides is stimulated when *T. aundinaceum* was grown at suboptimal conditions, e.g., 20°C, as was previously described for IBT 40837 strain (Cardoza et al., 2022). Furthermore, in correlation with their ability to produce HA, the three isolates also shown a significant antifungal activity against the bean pathogens *R. solani* and *S. sclerotiorum*, in comparison to the HA non-producer mutant Δtri5.3, which agrees with previous reports indicating the importance of HA production on the antifungal activity of the producer *Trichoderma* isolates (Malmierca et al., 2013).

Regarding plant growth, an especially significant positive effect on growth was observed in the growth of aerial parts, but only in the presence of TP6.6. Growth of root system was also increased in the presence of the same isolate but only in plants grown in substrate infected with *S. sclerotiorum* S47. Positive effects of *Trichoderma* inoculation on bean germination and growth had been already reported (Pereira et al., 2014; Mayo-Prieto et al., 2015). However, in the present case a great difference between both HA producers (TP19.13 and TP6.6) isolated in this work was observed, which would indicate that even though these isolates share a common origin some differences between them, e.g., level of HA production, would give also to different plant-responses. Therefore, for the use of *T. arundinaceum* isolates in biocontrol, strain selection should be essential to maximize their positive effects.

On the other hand, the absence of obvious “root rot” symptoms observed in the present work in plants infected with *R. solani* and *S. sclerotiorum*, could be attributed to the differences in temperature, substrate, humidity, etc, used in the incubation chambers to perform the current study, in comparison versus the conditions existing in the natural field environment. Thus, most likely the variations in environmental conditions might have been able to negatively influence the appearance of pathogenicity symptoms caused by *R. solani* and S*. sclerotiorum*

Furthermore, in order to characterize the importance of HA production to *Trichoderma*-bean interactions, a transcriptomic study was performed, which was later validated with a qPCR partial analysis. Thus, production of trichothecenes drastically affected expression of genes related to plant defense, being the highest differences observed when compared TP19.13 vs Δtri5.3 inoculated plants, which would indicate the remarkable effect exerted by trichothecenes in the bean defense response. In this sense TP19.13 down-regulated genes related to jasmonate, oxylipins, and ethylene signaling pathways in comparison versus plants inoculated with Δtri5.3, in both cases without pathogen. However, an opposite effect was observed when *S. sclerotiorum* was present. There are multiple reports on the effect of *Trichoderma* isolates on the transcriptome of bean plants, particularly related to effects on defense related genes (Lehtonen et al., 2008; Guerrero-González et al., 2011; Pereira et al., 2014; Mayo-Prieto et al., 2015; Mayo-Prieto et al., 2016). However, the results from the current study provide evidence that trichothecene production can impact *in vivo* on the transcription of defense related genes in *P. vulgaris*.

Four chitinase-encoding genes were upregulated by TP19.13 versus Δtri5.3, and only one chitinase was positively regulated at a higher level by Δtri5.3, which suggest that HA might induce plant-defense response, in agreement with previous reports (Malmierca et al., 2012; Malmierca et al., 2013), and also emphasizes the role of chitinases in the plant-defense response (Lehtonen et al., 2008; Lopes et al., 2012). However, the pattern of expression of other groups of genes was diverse, e.g., ethylene-related genes, with some genes up-regulated in plants inoculated with Δtri5.3, and others in plants with TP19.13.

In conclusion, the procedures used in the present study to recover trichothecene-producing *Trichoderma* strains from bean field soils, which were initially based on classical microbiological methods, and further supported by genomic, metabolomic and transciptomic analyses, have been successful. All isolates of *T. arundinaceum* recovered in this study produced HA, in addition to aspinolides, the latter being secondary metabolites with bioactivities similar to those of HA. The three bean-field isolates of *T. arundinaceum* exhibit different levels of HA production. Despite that they all exhibited a strong *in vitro* antifungal effect against *R. solani* and *S. sclerotiorum*. In addition, *T. arundinaceum* strains isolated from bean fields stimulated bean seed germination and promote growth of above-ground parts of the bean plants, the latter only in the case inoculated with isolate TP6.6. Finally, trichothecene production by *T. arundinaceum* affected expression of plant defense-related genes, including up-regulation of plant hormone-signaling related genes in the presence of *S. sclerotiorum* and of most of the chitinase encoding genes analyzed. Results included in the current study show the potential for *Trichoderma* isolates that produce non-phytotoxic trichothecenes to induce plant defenses without negatively affecting germination and development.

## Data availability statement

The original contributions presented in the study are publicly available. The assembled Whole Genome Shotgun sequences have been deposited at DDBJ/ENA/GenBank under the accession numbers JAMPTP000000000 (for TP6.6), JAMQRS000000000 (TP15.11), and JAMQTF000000000 (TP19.13).

## Author contributions

SG and PAC conceived and designed the research. REC performed all genetic experiments and quantified harzianum A production. SPM conducted the GC-MS analyses to identify and quantify the metabolites produced by the different isolates and contributed to the final correction of the manuscript. SM-P, PAC, ÁR-G, AL, GC-H, and MPC performed the fungal-plant *in vivo* assays, and analyzed the results obtained from these experiments. NM-R conducted qPCR experiments and analyzed transcriptomic data. RHP and SG conducted the sequence and phylogenetic analysis. SG, PAC, and RHP wrote the manuscript. All authors read and approved the manuscript.

## Funding

This work is a part of the Spanish I+D+i Grants RTI2018-099600-B-I00 and PID2021-123874OB-I00, financed by the MCIN/ AEI/10.13039/501100011033. GC-H was awarded with a Grant from the Ministry of Education, Culture, and Sport (Spain) (Grant number FPU15/04681). NM-R was awarded with a Grant from the Junta de Castilla y León (Spain) (ORDEN EDU/875/2021, July 13th, 2021).

## Acknowledgments

We acknowledge to ecological farmers Gabriel Alegre and Juan Carlos García from AGRELE (“Agricultores y Ganaderos Ecológicos de León”) for their collaboration to collect the soil samples. Finally, we also acknowledge to José Álvarez and Paula Vales from the University of León, and Crystal Probyn, Amy McGovern, and Christine Poppe from the United States Department of Agriculture for their excellent technical assistance.

## Conflict of interest

The authors declare that the research was conducted in the absence of any commercial or financial relationships that could be construed as a potential conflict of interest.

## Publisher’s note

All claims expressed in this article are solely those of the authors and do not necessarily represent those of their affiliated organizations, or those of the publisher, the editors and the reviewers. Any product that may be evaluated in this article, or claim that may be made by its manufacturer, is not guaranteed or endorsed by the publisher.

## Author disclaimer

Mention of trade names or commercial products in this publication is solely for the purpose of providing specific information and does not imply recommendation or endorsement by the U.S. Department of Agriculture. USDA is an equal opportunity provider and employer.
