# Supplementary material for: Effects of trichothecene production by Trichoderma arundinaceum isolates from bean-field soils on the defense response, growth and development of bean plants (Phaseolus vulgaris)
Source: Front Plant Sci. 2022 Nov 14;13:1005906. doi: 10.3389/fpls.2022.1005906 (PMC9702529; doi:10.3389/fpls.2022.1005906)
Supplement: Supplementary file 1 [file DataSheet_1.pdf]

Supplementary material

Effects of trichothecene production by *Trichoderma arundinaceum* isolates from bean-field soils on the defense response, growth and development of bean plants (*Phaseolus vulgaris*)

Cardoza, Rosa E.[1]; Mayo-Prieto, Sara[2]; Martínez-Reyes, Natalia[1]; McCormick, Susan P.[3]; Carro-Huerga, Guzmán[2]; Campelo, M. Piedad[2]; Rodríguez-González, Álvaro[2]; Lorenzana, Alicia[2]; Proctor, Robert H.[3]; Casquero, Pedro A.[2]*; Gutiérrez, Santiago[1]*

1.- University Group for Research in Engineering and Sustainable Agriculture (GUIIAS), Area of Microbiology, Universidad de León, Ponferrada, Spain
2.- University Group for Research in Engineering and Sustainable Agriculture (GUIIAS), Area of Crop Production, Universidad de León, León, Spain.
3.- Mycotoxin Prevention and Applied Microbiology Research Unit, National Center for Agricultural Utilization Research, Agriculture Research Service, U.S. Department of Agriculture. Peoria, Illinois, United States.

Table S1. Results of the preliminary identification of the 113 isolates selected from the 28 soil samples analyzed

Sample*	Identification (Blastn/NCBI)**	Sample*	Identification (Blastn/NCBI)
**1.4**	***Trichoderma virens***	**13.4**	***Trichoderma spirale***
**1.7**	***Trichoderma virens***	**13.5**	***Trichoderma spirale***
**1.12**	***Trichoderma rosicum***	**13.6**	***Trichoderma spirale***
1.14	*Chaetomium madrasense*	13.9	*Fusarium oxysporum*
**2.8**	***Trichoderma virens***	14.2	*Clonostachys rosea*
**2.9**	***Trichoderma virens***	14.4	*Hypocreales* sp.
2.15	*Penicillium hordei*	14.13	*Papulaspora sepedonioides*
**3.2**	***Trichoderma hamatum***	**15.7**	***Trichoderma rossicum***
**3.4**	***Trichoderma koningiopsis***	**15.8**	***Trichoderma harzianum***
**3.5**	***Trichoderma harzianum***	**15.11**	***Trichoderma brevicompactum***
**3.6**	***Trichoderma spirale***	15.12	*Fusarium oxysporum*
**3.10**	***Trichoderma gamsii***	**16.3**	***Trichoderma virens***
**3.11**	***Trichoderma harzianum***	16.4	*Fusarium oxysporum*
**3.11-2**	***Trichoderma spirale***	**16.11**	***Trichoderma virens***
**3.15**	***Trichoderma harzianum***	**16.12**	***Trichoderma virens***
**4.1**	***Trichoderma harzianum***	16.13	*Fusarium oxysporum*
**4.2**	***Trichoderma harzianum***	17.7	*Penicillium griseofulvum*
**4.3**	***Trichoderma harzianum***	**18.1**	***Trichoderma koningii***
**4.13**	***Trichoderma hamatum***	18.2	*Fusarium oxysporum*
5.2	*Clonostachys rosea*	**18.3**	***Trichoderma harzianum***
**5.4**	***Trichoderma virens***	**18.4**	***Trichoderma gamsii***
**5.14**	***Trichoderma virens***	**18.6**	***Trichoderma gamsii***
**6.4**	***Trichoderma harzianum***	18.11	*Fusarium oxysporum*
**6.6**	***Trichoderma brevicompactum***	**19.2**	***Trichoderma rossicum***
**6.13**	***Trichoderma brevicompactum***	**19.4**	***Trichoderma rossicum***
**7.4**	***Trichoderma harzianum***	19.6	*Fusarium solani*
**7.12**	***Trichoderma harzianum***	**19.9**	***Trichoderma rossicum***
**8.11**	***Trichoderma virens***	**19.10**	***Trichoderma rossicum***
**8.15**	***Trichoderma velutinum***	19.11	*Mucor circinelloides*
**8.19**	***Trichoderma spirale***	**19.13**	***Trichoderma brevicompactum***
**8.22**	***Trichoderma erinaceum***	**19.14**	***Trichoderma gamsii***
**8.23**	***Trichoderma harzianum***	19.15	*Fusarium solani*
**9.12**	***Trichoderma paraviridescens***	**19.16**	***Trichoderma tomentosum***
**9.14**	***Trichoderma velutinum***	**19.18**	***Trichoderma tomentosum***
**9.15**	***Trichoderma velutinum***	**19.19**	***Trichoderma rossicum***
**10.2**	***Trichoderma velutinum***	**19.22**	***Trichoderma harzianum***
**10.4**	***Trichoderma velutinum***	**19.24**	***Trichoderma rossicum***
**10.5**	***Trichoderma velutinum***	**19.25**	***Trichoderma harzianum***
**10.6**	***Trichoderma virens***	20.1	*Fusarium oxysporum*
**10.12**	***Trichoderma tomentosum***	20.2	*Fusarium oxysporum*
**10.13**	***Trichoderma velutinum***	**21.1**	***Trichoderma gamsii***
**10.14**	***Trichoderma velutinum***	**21.5**	***Trichoderma gamsii***
**10.15**	***Trichoderma velutinum***	**22.1**	***Trichoderma harzianum***
**10.16**	***Trichoderma harzianum***	**22.6**	***Trichoderma harzianum***
**10.17**	***Trichoderma velutinum***	**22.7**	***Trichoderma harzianum***

**10.18**	***Trichoderma harzianum***	23.5	*Clonostachys rosea*
**10.20**	***Trichoderma harzianum***	24.1	*Fusarium proliferatum*
11.5	*Mucor circinelloides*	24.13	*Fusarium solani*
**11.6**	***Trichoderma gamsii***	25.7	*Fusarium oxysporum*
**11.7**	***Trichoderma gamsii***	25.12	*Fusarium proliferatum*
**12.2**	***Trichoderma tomentosum***	25.13	*Fusarium solani*
**12.3**	***Trichoderma hamatum***	**26.1**	***Trichoderma harzianum***
**12.6**	***Trichoderma virens***	**26.7**	***Trichoderma harzianum***
12.7	*Penicillium chrysogenum*	27.1	*Penicillium annulatum*
**13.3**	***Trichoderma virens***	**27.8**	***Trichoderma virens***
		**27.10**	***Trichoderma harzianum***
		27.12	*Fusarium oxysporum*
		28.4	*Fusarium oxysporum*

*Sample code.- Number before the dot corresponds to the number assigned to each field-soil sample (1-28), and second number after the dot corresponds to a particular isolate among the total isolates analyzed from a particular soil. For example, sample 27.12 corresponds to the isolate number 12 among those obtained from the soil sample number 27.

**Note that those *Trichoderma* isolates preliminary identified as corresponding to clade Brevicompactum have been shaded in yellow. *Fusarium* spp. were written in green letters, and blue colored letters were used for non-*Trichoderma*, non-*Fusarium* identified isolated.

Table S2. Genomic sequences of *Trichoderma* spp. used to retrieve *tri5* genes to be used in the phylogenetic analyses carried out in the current study.

Species	GenBank accession number	Reference
Trichoderma asperelloides T203	GCA_021066465.1	Gortikov,M., Wang,Z., Steindorff,A.S., Grigoriev,I.V.,Druzhinina,I.S., Townsend,J.P. and Yarden,O. 2021. Sequencing and analysis of the entire genome of the mycoparasitic fungus *Trichoderma asperelloides* strain T203. Unpublished.
Trichoderma erinaceum CRRI-T2N1	GCA_013365115.1	Mukherjee, A.K. and Swain,H. 2021. Whole genome sequence of *Trichoderma erinaceum*. Unpublished
Trichoderma simmonsii GH-Sj1	GCA_019565615.1	Chung, S., Kwon, Y.M., Yang, Y. 2021. Telomere-to-telomere genome assembly of asparaginase-producing *Trichoderma simmonsii*. BMC Genomics. 22: 830. doi: 10.1186/s12864-021-08162-4.
Trichoderma sp. IMV 00454	GCA_001931985.1	Venkateswaran, k. 2016. Draft genome sequences of several fungal strains selected for exposure to microgravity at the international space station. Unpublished.
Trichoderma cornu-damae KA19-0412C	GCA_020631695.1	Kim, C.S. 2021. Chromosome-level *Trichoderma cornu-damae* using Hi-C data. Unpublished

Table S3.
Seeds Germination. Probability values (two-way ANOVA) for Pathogen and *Trichoderma* treatments on bean seed germination.

Source of variation	Germination			
	9 days	12 days	18 days	25 days
Pathogen	0.576	0.701	0.835	0.675
Trichoderma	0.000	0.000	0.218	0.259
Pathogen x *Trichoderma*	0.213	0.221	0.141	0.028

Table S4.
Plant Growth. Probability values (two-way ANOVA) for Pathogen and *Trichoderma* treatments on bean plant growth.

Source of variation	Dry weight (g) of the aerial part	Dry weight (g) of the root system
Pathogen	0.360	0.095
Trichoderma	0.038	0.462
Pathogen x *Trichoderma*	0.201	0.024

Table S5. Trimmed data stats. Total number of trimmed bases, reads, GC (%), and Q30 calculated for the 18 samples analyzed.

***T. arundinaceum* strain**	**Plant Pathogen**	**Biological Replicate**	**Total read bases**	**Total reads**	**GC (%)**	**Q30 (%)**
None (=CC)	None	1	10,401,641,998	70,787,612	45.41	95.84
∆T5.3	None	1	10,914,049,260	74,242,600	45.21	96.23
TP19.13	None	1	9,826,921,071	66,360,214	44.78	96.04
None (=CR)	*R. solani*	1	10,160,970,188	69,729,470	44,18	95.99
∆T5.3	*R. solani*	1	10,810,039,213	73,843,096	44.74	96.05
TP19.13	*R. solani*	1	9,952,151,244	68,478,022	45.38	95.78
None (=CS)	*S. sclerotiorum*	1	10,190,296.625	70,206,284	45.45	96.29
∆T5.3	*S. sclerotiorum*	1	10,359,056,986	70,629,488	44.00	96.07
TP19.3	*S. sclerotiorum*	1	9,450,262,666	64,346,976	44.84	96.03
None (=CC)	None	2	9,839,681,706	67,224,504	45.96	95.97
∆T5.3_2	None	2	10,001,360,290	68,445,604	45.21	95.98
TP19.13	None	2	10,313,581,381	70,651,002	45.08	96.00
None (=CR)	*R. solani*	2	9,750,697,994	67,285,196	44.76	96.16
∆T5.3	*R. solani*	2	10,157,001,180	69,010,642	45.22	95.97
TP19.13	*R. solani*	2	10,389,105,342	71,369,874	45.35	95.95
None (=CS)	*S. sclerotiorum*	2	9,926,015,181	68,179,608	45.00	96.17
∆T5.3	*S. sclerotiorum*	2	9,933,566,320	67,960,886	43.91	96.08
TP19.13	*S. sclerotiorum*	2	8,789,310,322	60,203,664	44.59	96.20

Table S6. Filtered number of differentially expressed genes in the 9 comparisons analyzed in the present work.

**Compared conditions**	**Number of DEG***
**ΔT5 vs CC**	924
**19 vs CC**	1,194
**19 vs ΔT5**	1,570
**ΔT5R vs CR**	755
**19R vs CR**	1,677
**19R vs ΔT5R**	715
**ΔT5S vs CS**	3,786
**19S vs CS**	1,745
**19S vs ΔT5S**	2,576
**Total**	**14,942**

* DEG.- differentially expressed genes.
Note that Statistical analysis, carried out using Fold Change per comparison pair, was performed with data that have been filtered to remove the low-quality transcripts. The significant results are selected on conditions of fold change /fc/>=2 & exactTest raw *p*-value<0.05.

Table S7. Defense-related genes of *Phaseolus vulgaris* used for qPCR analysis.

Gene	Predicted function	Functional Group	Reference
CHS.3	Chalcone synthase	Phenylpropanoid biosynthesis	Mayo et al., 2016
CPRD14.4	CPRD14 protein	Stress response	Mayo et al., 2016
CPRD14.3	CPRD14 protein	Stress response	Mayo et al., 2016
Lox2.3	Lipooxygenase	Antimicrobials and oxylipins (defense signaling molecules)	Porteous-Álvarez et al., 2020
Lox1.3	Lipooxygenase	Antimicrobials and oxylipins (defense signaling molecules)	Pereira et al., 2014
AOS.4	Allene oxide synthase	Oxylipin pathway	Mayo et al., 2016
LTP2.1	Lipid transfer protein 2	Pathogenesis related genes	Mayo et al., 2016
MMP2.2	Matrix metalloproteinase 2	Pathogenesis related genes	Mayo et al., 2016
TSI-1.2	TSI-1 protein (Pathogenesis related 10)	Pathogenesis related genes	Mayo et al., 2016
PR1.2	Pathogenesis related proteins 1 and 2	Pathogenesis related genes	Porteous-Álvarez et al., 2020
PR16a	Germin like protein 10 (Pathogenesis related 16a)	Pathogenesis related genes	Porteous-Álvarez et al., 2020
GTSa.1	2,4-Glutathione S-transferase 1	Oxidative stress	Mayo et al., 2016
Ch5b.3	Endochitinase precursor	Pathogenesis related genes	Porteous-Álvarez et al., 2020
ERF5	Ethylene-responsive Transcription factor 5	Ethylene signaling pathway	Porteous-Álvarez et al., 2020
SIP.3	Syringolide induced protein B13-1-9	Defense	Mayo et al., 2016

Mayo-Prieto, S., Cominelli, E., Sparvoli, F., González-López, O., Rodríguez-González, A., Gutiérrez, S., Casquero, P.A. (2016). Development of a qPCR strategy to select bean genes involved in plant defense response and regulated by the *Trichoderma velutinum- Rhizoctonia solani* interaction. *Front. Plant Sci.* 7, 1109. doi: 10.3389/fpls.2016.01109.

Pereira, J.L., Queiroz, R.M.L., Charneau, S.O., Felix, C.R., Ricart, C.A.O., Lopes da Silva, F., Steindorff, A.S., Ulhoa, C.J., Noronha, E.F. (2014). Analysis of *Phaseolus vulgaris* response to its association with *Trichoderma harzianum* (ALL-42) in the presence of absence of the phytopathogenic fungi *Rhizoctonia solani* and *Fusarium solani*. *PLoS ONE* 9, e98234. doi: 10.1371/journal.pone.0098234

Porteous-Álvarez, A.J., Mayo-Prieto, S., Álvarez-García, S., Reinoso, B., Caquero, P.A. (2020). Genetic response of common bean to the inoculation with indigenous *Fusarium* isolates. *J. Fungi* 6, 228. doi: 10.3390/jof6040228.

Table S8. Oligonucleotides used for qPCR analysis

Gene name	Gene code	Primer name	Primer sequence (5´-3´)	Primer pairs amplification efficiency (%)	Amplicon size (bp)
CHS.3	PHAVU_002G184300g	CHS_3_F	TGTGCTTGTGGTGTGTTCTG	86.6	107
		CHS_3_R	CAGCTGCTCCATCTCCAAAC		
CPRD14.4	PHAVU_L005700g	CPRD14_4_F	ACACAGCACATTTCAACGCT	85.4	129
		CPRD14_4_R	AGGAGAAACTTCACGAGCCA		
CPRD14.3	PHAVU_005G034700g	CPRD14_3_F	CGCTGGACAAGTAGTGTGTG	85.5	66
		CPRD14_3_R	AGAAACTTGACGAGCCAGGA		
Lox2.3	PHAVU_010G134900g	Lox2_3_F	AACTGGGCAGCGTAAGGTAT	91.3	106
		Lox2_3_R	ACCTCCAAGAACAGGACGAG		
Lox1.3	PHAVU_010G135000g	lox1_3_F	GGCCTACCCAACACAAAGTG	100.0	108
		lox1_3_R	TCCCTCAGCATGTGGTAGAC		
AOS.4	PHAVU_003G010700g	AOS_4_F	ACGGAGGAACCTTCTGTGAG	90.9	143
		AOS_4_R	ACACTTGGACCAAACCCAGA		
LTP2.1	PHAVU_004G002700g	LTP2_1_F	CGGAGCTGAGTCCTTGTTTG	92.9	88
		LTP2_1_R	CATGGCCTTTGTTCCCTCAC		
MMP2.2	PHAVU_002G076700g	MMP2_2_F	GACCTTCCAACGCTCTTTCC	107.8	80
		MMP2_2_R	ATCGTGGTCGCGAGATTAGT		
TSI-1.2	PHAVU_009G252300g	TSI-1_2_F	GTGATGGTGGTTGCCTTTGT	97.7	142
		TSI-1_2_R	GGATTCTGAAGCAGGTAGGC		
PR1.2	PHAVU_003G109300g<br>PHAVU_003G109500g<br>PHAVU_003G109800g<br>PHAVU_003G109600g	PR1-2_F	ATAGTTGGAGGTGCTGCCTT	86.8	127
		PR1-2_R	GAGCATCTCCTTTGCTGTGG		
PR16a	PHAVU_010G129900g	PR16a_F	TTCTGTGTGGCAGACCTCAA	114.4	145
		PR16a_R	GGGACACAGCAGCATTGATT		
GTSa.1	PHAVU_005G054100g	GTSa_1_F	GGGCAAGCCCATTTCTGAAT	89.3	104

		GTSa_1_R	CCTGAGCTCTCTGGTAAGGG		
CH5b.3	PHAVU_009G116300g	CH5b_3_F	GCCATTGGGCTTGACTTGAT	97.3	139
		CH5b_3_R	TCCATCGAGAGGTGATGACG		
ERF5	PHAVU_002G055700g	ERF5_F	GGCAAGTGGGAAGTCAAGTG	86.0	144
		ERF5_R	CACTGTCACACGCATCTCAG		
SIP.3	PHAVU_001G194900g	SIP_3_F	GCAATACACGAAGAGCAGCA	91.7	110
		SIP_3_R	CGCCATCACTCTTGTCTTGG		
actin	PHAVU_008G011000g	Act_F	CTCTCCCTCTTTGCCTTCCA	88.3	134
		Act_R	ATCTCCAGCAAATCCAGCCT		

A

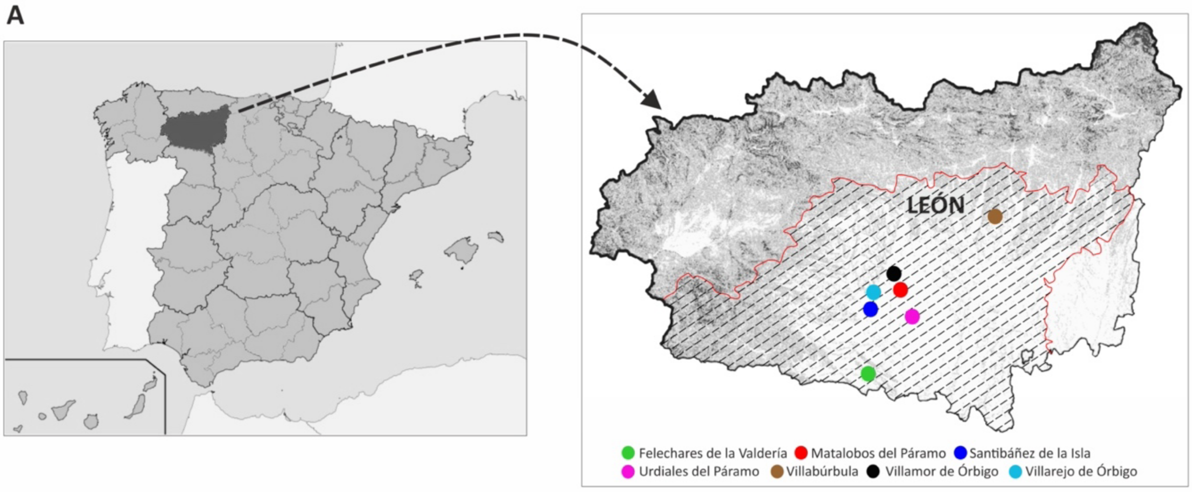

B

Sample	Location (County)	Coordinates	Date of collection month/day/year
1	Bustillo del Páramo	42° 27' 40" N 5° 47' 45" W	10/08/2019
2	Villarejo de Órbigo	42° 26' 42" N 5° 53' 42" W	10/08/2019
3	Felechares de la Valdería	42° 11' 23" N 6° 2' 42" W	10/09/2019
4	Villamor de Órbigo	42° 28' 46" N 5° 52' 22" W	10/09/2019
5	Matalobos del Páramo-1	42° 24' 53" N 5° 50' 5" W	10710/2019
6	Matalobos del Páramo-2	42° 24' 54" N 5° 49' 59" W	10/10/2019
7	Matalobos del Páramo-3	42° 24' 40" N 5° 49' 43" W	10/11/2019
8	Matalobos del Páramo-4	42° 24' 30" N 5° 49' 42" W	10/11/2019
9	Urdiales del Páramo-1	42° 24' 17" N 5° 49' 41" W	10/14/2019
10	Urdiales del Páramo-2	42° 24' 51" N 5° 49' 28" W	10/14/2019
11	Santibáñez de la Isla-1	42° 22' 38" N 5° 55' 8" W	10/15/2019
12	Santibáñez de la Isla-2	42° 22' 54" N 5° 56' 2" W	10/15/2019
13	Santibáñez de la isla-3	42° 23' 8" N 5° 56' 12" W	10/15/2019
14	Matalobos del Páramo-5	42° 25' 12" N 5° 48' 47" W	02/03/2020
15	Matalobos del Páramo-6	42° 25' 10" N 5° 48' 46" W	02/03/2020
16	Matalobos del Páramo-7	42° 25' 13" N 5° 48' 40" W	02/03/2020
17	Matalobos del Páramo-8	42° 25' 4" N 5° 48' 9" W	02/03/2020
18	Matalobos del Páramo-9	42° 25' 12" N 5° 48' 0" W	02/03/2020
19	Matalobos del Páramo-10	42° 25' 10" N 5° 47' 53" W	02/03/2020
20	Matalobos del Páramo-11	42° 24' 51" N 5° 48' 57" W	02/03/2020
21	Villabúrbula-1	42° 33' 44" N 5° 26' 4" W	02/03/2020
22	Villabúrbula-2	42° 33' 43" N 5° 26' 5" W	02/04/2020
23	Villabúrbula-3	42° 33' 45" N 5° 26' 9" W	02/04/2020
24	Villabúrbula-4	42° 33' 45" N 5° 26' 10" W	02/04/2020
25	Villabúrbula-5	42° 33' 44" N 5° 26' 10" W	02/04/2020
26	Villabúrbula-6	42° 33' 43" N 5° 26' 11" W	02/04/2020
27	Villabúrbula-7	42° 33' 42" N 5° 26' 5" W	02/04/2020
28	Villabúrbula-8	42° 33' 41" N 5° 26' 6" W	02/04/2020

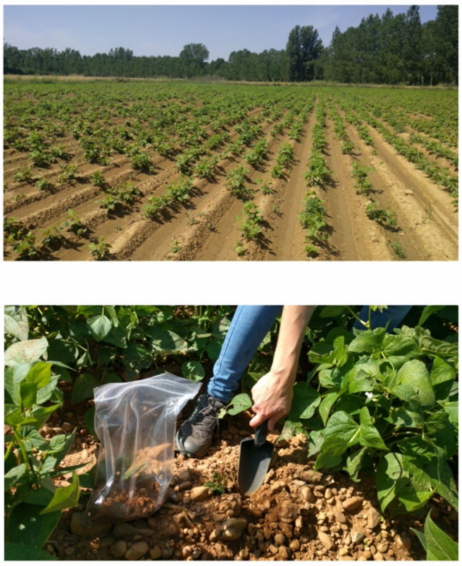

Figure S1. A. <u>Left panel</u>: Localization of the Province of León (Spain). **<u>Right panel</u>**: The localization of the Protected Geographic Indication (PGI) “Alubia de La Bañeza-León” corresponds to the red-delimitated area labeled with dashed-lines. Soil samples used in the present work were collected from fields located at the municipalities indicated with colored spots. **B. <u>Left panel</u>**: Table indicating fields used to collect the soil samples, geographical coordinates of the different plots, and the date of samples collection. Note that different number associated to the same municipality name indicated different fields from that municipality used to collect the samples. **<u>Right panel</u>**: Photographs illustrating the aspect of a bean crop in the moment to collect the samples (up) and the moment when a sample was collected (down).

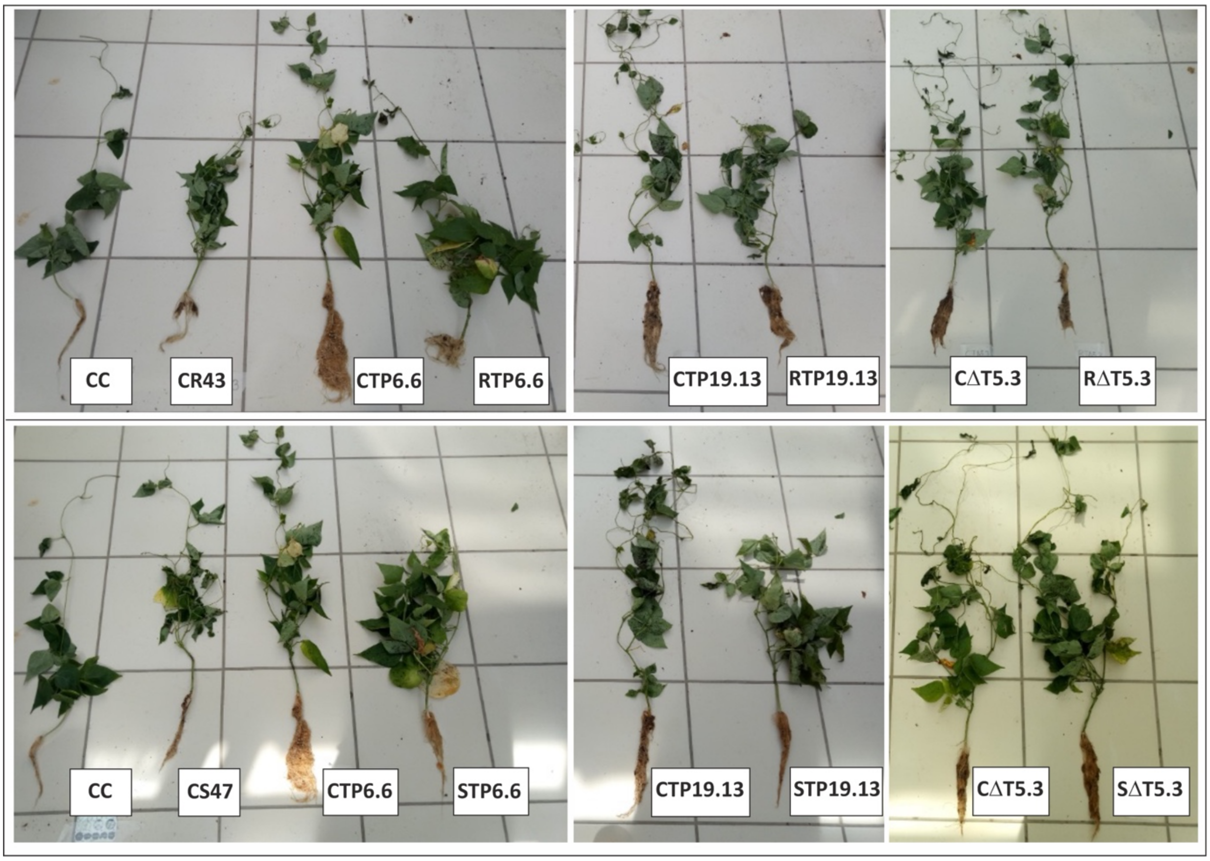

Figure S2. Photographs including washed plants after 45 days of growth from the 12 treatments performed in the present study. CC= bean plants grown without *Trichoderma* inoculation in substrate free of pathogens. CR43 and CS47 correspond to plants grown in substrate infected with the pathogens *R. solani* R43 and *S. sclerotiorum* S47, respectively. CΔT5.3, CTP6.6, and CTP19.13= plants grown from seeds inoculated with spores of ΔT5.3, TP6.6, and TP19.13, respectively, without previous pathogen inoculation. R# and S#= plants grown from seeds coated with the *Trichoderma* strain indicated on each name (#=ΔT5.3, TP6.6, or TP19.13), in a substrate previously infected with *R. solani* R43 (R#) and *S. sclerotiorum* S47 (S#), respectively.

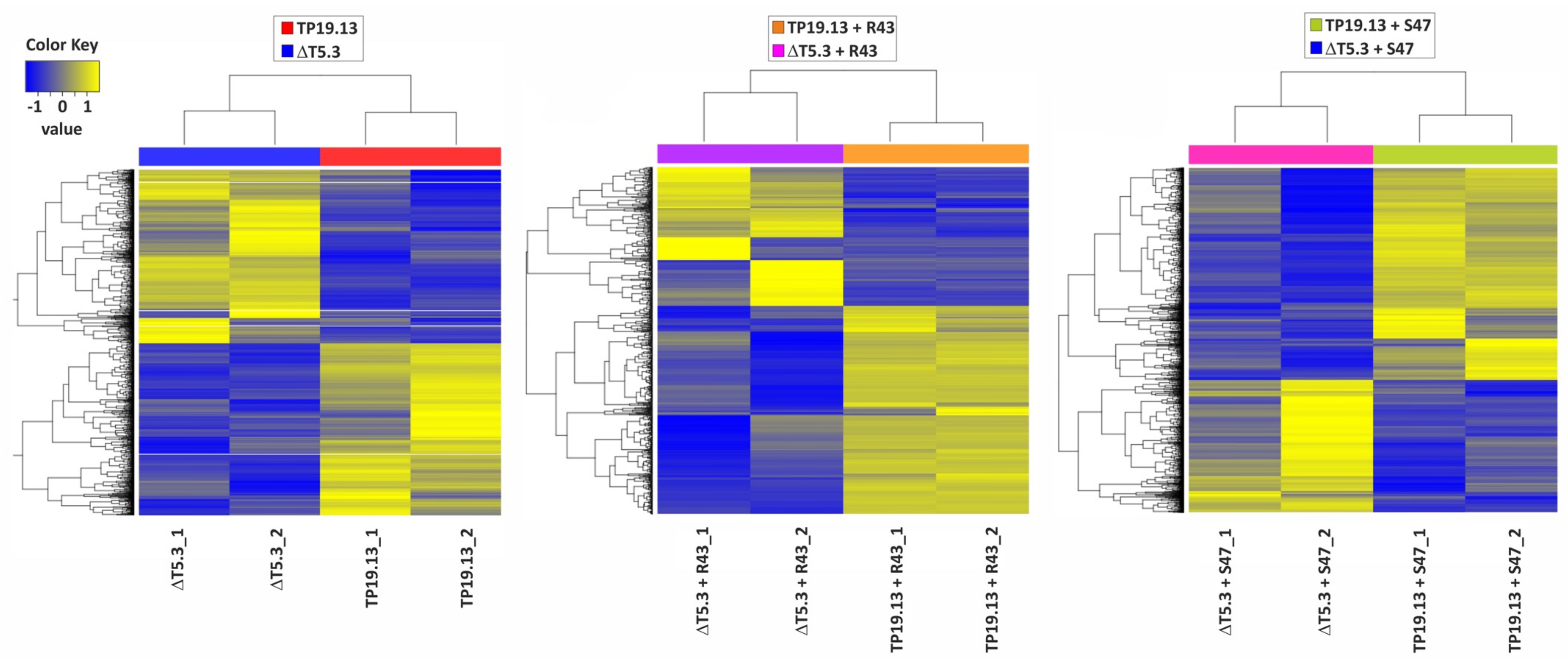

Figure S3. Heat map representing the *Phaseolus vulgaris* differential gene expression in the comparisons TP19.13 vs ΔT5.3 (left panel), TP19.13+R43 vs ΔT53+R43 (central panel), and TP19.13+S47 vs ΔT5.3+S47 (right panel) of the one-way Hierarchical Clustering using Z-score for normalized value (log2 based) (1,570/ 715/ and 2,576 transcripts, respectively, satisfying with fold change /fc/ >=2 and exact Test raw *p*-value<0.05).
